# A Germline Heterozygous Dominant Negative *IKZF2* Variant Causing Syndromic Primary Immune Regulatory Disorder and ICHAD

**DOI:** 10.1007/s10875-025-01882-2

**Published:** 2025-04-28

**Authors:** Henry Y. Lu, Maryam Vaseghi-Shanjani, Avery J. Lam, Mehul Sharma, Arezoo Mohajeri, Leandro B. R. Silva, Jana Gillies, Gui Xiang Yang, Susan Lin, Maggie P. Fu, Areesha Salman, Ronak Rahmanian, Linlea Armstrong, Jessica Halparin, Connie L. Yang, Mark Chilvers, Erika Henkelman, Wingfield Rehmus, Douglas Morrison, Audi Setiadi, Sara Mostafavi, Michael S. Kobor, Frederick K. Kozak, Catherine M. Biggs, Clara van Karnebeek, Kyla J. Hildebrand, Anna Lehman on behalf of the Care4Rare Canada Consortium, Megan K. Levings, Stuart E. Turvey

**Affiliations:** 1 Department of Pediatrics, BC Children’s Hospital, The University of British Columbia, 950 West 28 th Avenue, Vancouver, BC V5Z 4H4 Canada; 2 Experimental Medicine Program, Faculty of Medicine, The University of British Columbia, Vancouver, BC Canada; 3 BC Children’s Hospital Research Institute, Vancouver, BC Canada; 4 Department of Surgery, The University of British Columbia, Vancouver, BC Canada; 5 Department of Medical Genetics, The University of British Columbia, Vancouver, BC Canada; 6 Genome Science and Technology Program, Faculty of Science, The University of British Columbia, Vancouver, BC Canada; 7 Centre for Molecular Medicine and Therapeutics, Vancouver, BC Canada; 8 Division of Otolaryngology – Head & Neck Surgery, BC Children’s Hospital, The University of British Columbia, Vancouver, BC Canada; 9 Division of Hematology, Oncology & Bone Marrow Transplant, BC Children’s Hospital, The University of British Columbia, Vancouver, BC Canada; 10 Division of Respiratory Medicine, BC Children’s Hospital, The University of British Columbia, Vancouver, BC Canada; 11 Division of Plastic Surgery, BC Children’s Hospital, The University of British Columbia, Vancouver, BC Canada; 12 Division of Dermatology, BC Children’s Hospital, The University of British Columbia, Vancouver, BC Canada; 13 Department of Pathology and Laboratory Medicine, BC Children’s Hospital, The University of British Columbia, Vancouver, BC Canada; 14 Department of Statistics, The University of British Columbia, Vancouver, BC Canada; 15 Division of Immunology, BC Children’s Hospital, The University of British Columbia, Vancouver, BC Canada; 16 School of Biomedical Engineering, The University of British Columbia, Vancouver, BC Canada

## Abstract

Monogenic defects that impair the control of inflammation and tolerance lead to profound immune dysregulation, including autoimmunity and atopy. Studying these disorders reveals important molecular and cellular factors that regulate human immune homeostasis and identifies potential precision medicine targets. Here, we provide a detailed immunological assessment of a pediatric patient with a recently discovered syndrome causing Immunodysregulation, Craniofacial anomalies, Hearing impairment, Athelia, and Developmental delay (or ICHAD syndrome). The immunodysregulation resulted in autoimmune hemolytic anemia (AIHA) and atopic dermatitis. The patient carried a de novo germline heterozygous c.406+540_574+13477dup;p.Gly136_Ser191dup variant in *IKAROS family zinc finger 2* (*IKZF2*), which encodes HELIOS. This variant led to reduced HELIOS protein expression and dominant interference of wild-type HELIOS-mediated repression of the *IL2* promoter. Multi-parameter flow cytometry analyses of patient peripheral blood mononuclear cells revealed strongly impaired natural killer cell differentiation and function, and increased CD8^+^ T cell activation and cytokine secretion. Strikingly, patient CD4^+^ T cells were hyperactive, produced elevated levels of nearly all T helper (T_H_) cytokines, and readily proliferated in response to stimulation. Patient regulatory T cells (Tregs) developed normally but aberrantly produced high levels of many T_H_ cytokines. Single-cell RNA sequencing revealed largely normal Tregs (albeit mostly memory), but naïve CD4^+^ T cells that were more enriched in genes related to activation, proliferation, metabolism, and T_H_ differentiation. This work describes the immunological phenotype of one of the first reported cases of germline dominant negative HELIOS deficiency, expands our understanding of the pathogenesis of AIHA on a single cell level, and provides valuable insights into HELIOS function in a variety of lymphocyte subsets.

**Supplementary Information:**

The online version contains supplementary material available at 10.1007/s10875-025-01882-2.

## Introduction

Immunity is tightly regulated to optimize protective responses against pathogens and malignancy while preventing inappropriate responses to otherwise benign antigens. Monogenic defects that impair this regulation of inflammation or tolerance result in a subgroup of the inborn errors of immunity (IEI) called primary immune regulatory disorders (PIRD) [1]. In contrast to classic IEIs, which typically manifest as unusually severe or recurrent infections, PIRDs predominantly present with immune-mediated pathology, including autoimmunity, lymphoproliferation, malignancy, autoinflammation, and atopy, with susceptibility to infections being a less pronounced aspect of these disorders [2].

Immune dysregulation can lead to a variety of hematologic manifestations and cytopenias [3]. A classic example is autoimmune hemolytic anemia (AIHA) caused by immune-mediated destruction of erythrocytes [4]. AIHA can be idiopathic (primary) or secondary and is classified as warm or cold (cold agglutinin disease [CAD], paroxysmal cold hemoglobinuria) depending on autoantibody behaviour at different thermal ranges, which is important for determining appropriate treatment regimens [5]. Most cases of AIHA are considered idiopathic (~ 60%) [6], while secondary causes can include IEIs/PIRDs, malignancy, bacterial/viral infections, and drugs [4]. As such, the etiology and pathogenesis of AIHA remains incompletely understood.

Human germline variants in a family of transcription factors called the IKAROS zinc finger (IKZF) family have been linked to immunodeficiency and cytopenias [7–17]. Notably, loss-of-function (LOF) variants in *IKZF2* (HELIOS) were recently associated with combined immunodeficiency (CID) and/or immune dysregulation, including immune thrombocytopenia (ITP), Evan’s syndrome, and systemic lupus erythematosus [9–11]. *IKZF2* is highly expressed in hematopoietic stem/progenitor cells, T cells, including activated CD4^+^/CD8^+^ T cells, mucosa-associated invariant T (MAIT) cells, regulatory T cells (Tregs), and natural killer (NK) cells [18–23]. As Tregs are among the highest *IKZF2* expressors, most studies have focused on the role of HELIOS in Treg development and function [24–34]. In mice, Helios is critical for Treg identity, survival, and stability, but is seemingly dispensable in mice and humans for suppressive function [27, 34]. Nevertheless, both *Ikzf2*^*−/−*^ and Treg-specific *Ikzf2*^*−/−*^ mice develop progressive autoimmune disease associated with activated CD4^+^/CD8^+^ T cells, increased T follicular helper (T_FH_) and germinal center (GC) B cell numbers, autoantibody production, and increased proinflammatory cytokine production [26, 27, 35]. The role of *IKZF2* in other components of the human immune system (e.g. NK cells) is beginning to be better defined through the identification of humans with germline *IKZF2* variants [9–12].

Here, we report a detailed immunological and mechanistic workup of the first reported case of germline heterozygous dominant negative (DN) *IKZF2* variants in a young girl we recently described [12]. This patient was found to have a novel genetic syndrome comprising immunodysregulation, craniofacial anomalies, hearing impairment, athelia, and developmental delay (or ICHAD syndrome). Here we expand the understanding of ICHAD syndrome, reporting a comprehensive mechanistic immune evaluation of this patient which revealed significant developmental and functional defects in NK and naïve CD4^+^ T cells and marked immune activation as the likely cause of immune dysregulation. Informed by our improved appreciation of the human phenotype caused by germline *IKZF2* variants, we suggest that germline heterozygous dominant negative *IKZF2* variants should now be considered in the differential diagnosis of patients with AIHA and PIRD.

## Methods

### Study Participants and Consent

All study participants and/or their parents/guardians provided written informed consent. Research study protocols (H18-02853, H18-02912, H15-00092) were approved by The University of British Columbia Clinical Research Ethics Board.

### Cell Isolation, Culture, and Immortalization

Peripheral blood mononuclear cells (PBMCs) were isolated from all study participants by standard Ficoll-Paque (GE Healthcare) density centrifugation as previously described [36]. Lymphoblastoid cell lines (LCLs) were derived by standard EBV transformation as previously described [36] (Supplement).

### Plasmid Generation and Cloning

WT *IZKF2* and p.Gly136_Ser191dup *IKZF2* plasmids were generated by introducing restriction sites into cDNA or genomic DNA by PCR and cloning into pCMV6-XL4-3xFLAG vectors (Supplement).

### Transient Transfections

HEK293 cells were transfected with empty vector (EV), WT, p.Gly136_Ser191dup, or varying ratios of WT/p.Gly136_Ser191 dup *IKZF2* expression plasmids by Lipofectamine 3000 (ThermoFisher) transfection. In some experiments, cycloheximide (50 mg/mL) was added to the cells for 0, 6, 12, and 24 h, after initial incubation to assess protein stablity (Supplement). Transfected cells were then processed for immunoblotting or immunofluorescence.

### Immunoblotting

Endogenous HELIOS protein and FLAG-tagged HELIOS protein detection was accomplished in LCLs and transfected HEK293 cells by standard immunoblotting as previously described [36] (Supplement).

### Immunofluorescence

12 mm coverslips were sterilized, HEK293 cells were seeded on top in 6-well plates, allowed to attach for 24 h, then transfected as above. Transfected cells were fixed, permeabilized, and stained for HELIOS, DAPI, and F-actin before mounting and imaging on a Leica SP5 II Laser Scanning Confocal Microscope (Leica Microsystems GmbH) (Supplement).

### Immunophenotyping

Immunophenotyping and intracellular cytokine detection was performed as previously described [37]. Briefly, PBMCs were stimulated with PMA and ionomycin (P/I) in the presence of GolgiStop (#554724, BD Biosciences), stained with antibody panels 1–4 (Supplemental Table 5) using the eBioscience Foxp3 Transcription Factor Staining Buffer Set (#00–5523-00, Invitrogen, ThermoFisher), acquired on a FACSymphony flow cytometer, and analyzed using FlowJo (both BD Biosciences) (Supplement).

### Proliferation Assays

PBMCs were seeded at varying concentrations, labelled with cell proliferation dye (CPD) eF450 (#65–0842-85, Invitrogen, ThermoFisher) and stimulated with anti-CD3/CD28-coated Dynabeads (#11141D, Human T-Expander, Gibco, ThermoFisher) for four days. Cells were stained with antibody panel 6 (Supplemental Table 5) and acquired on a CytoFLEX (Beckman Coulter) and analyzed using FlowJo (Supplement).

### T Cell Suppression Assays

Treg suppression of CD3^+^ T cell proliferation was assessed as previously described [34]. Briefly, patient and control PBMCs were enriched for CD4^+^ T cells, sorted using panel 7 (Supplemental Table 5) for Tregs and conventional T cells (Tconvs), expanded with IL-2 and irradiated mouse L cells for seven days, then cocultured with various ratios of CPD-labelled anti-CD3/CD28 Dynabead-stimulated enriched CD3^+^ T responder (Tresp) cells for four days. Percent suppression of CD4^+^ and CD8^+^ T cell proliferation was calculated using division index (DI): (1—[DI of sample/DI of positive control]) × 100% (Supplement).

### Quantification of Cytokine Production

Supernatants were collected from PBMCs stimulated for proliferation, cocultured Tregs and Tresp for T cell suppression assays, and stimulated Tregs and Tconvs alone. Cytokine concentrations were measured by LEGENDplex Human Th Cytokine Panel 12-plex (#741027, BioLegend) on a CytoFLEX (Beckman Coulter) and analyzed with Qognit software (BioLegend) according to manufacturer’s recommendations (Supplement).

### TSDR Methylation

Genomic DNA was isolated from expanded Tregs and Tconvs, bisulfite converted with an EZ DNA Methylation-Direct Kit (Zymo Research), PCR amplified, and pyrosequenced using a PyroMark Q96 MD (Qiagen) as previously described [34] (Supplement).

### Single-Cell RNA Sequencing

Single-cell RNA sequencing was performed on unstimulated and 4 h P/I-stimulated sorted Tregs and Tconv from P1 and two age-matched/sex-matched controls using the BD Rhapsody Single Cell platform (BD Biosciences) according to manufacturer’s recommendations and analyzed as previously described [37] (Supplement). Raw data are deposited on Gene Expression Omnibus under accession number GSE236159.

### Statistical Analysis

Given that this study relied on repeated measurements from the same patient, we applied mixed-effects models conducted in R (version 4.3.1) using"lmer"function from"lme4"package, in which we used the subject ID as a random effect to account for intra-patient correlation and variability. This approach enables a more accurate representation of repeated measures and controls for the potential lack of independence in the data.

## Results

### Clinical Case Presentation

**Fig. 1:**
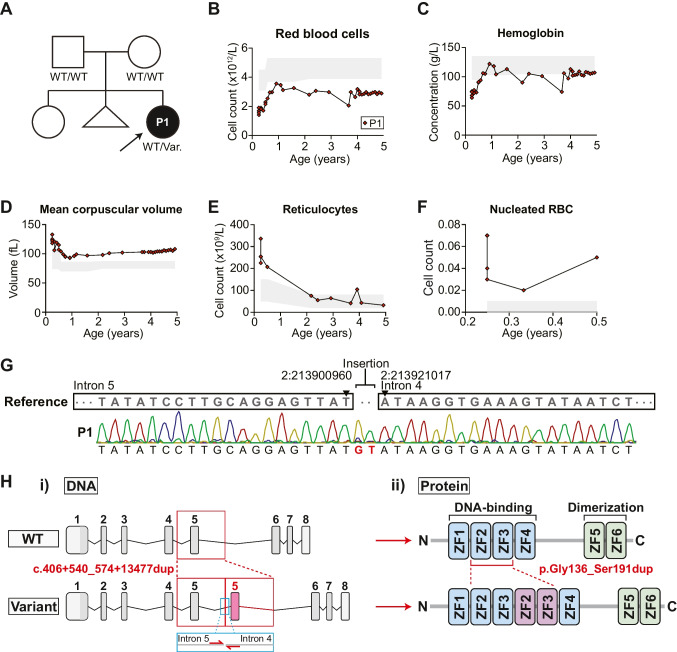
Clinical phenotype of the patient. **A** Family pedigree of the patient. Filled symbols indicate affected individuals. Arrow represents index patient. Known genotype is marked. **B**-**F** Hematological parameters measured in the patient over time. Shaded regions = age-specific reference ranges. **G** Sanger sequencing of DNA extracted from whole blood of the patient. Primers were designed to flank the breakpoint. **H** Schematic representation of the impact of i) the c.406+540_574+13477dup *IKZF2* variant on the genomic level. Red inset = exon 5 duplication. Blue inset = Sanger sequencing primer location; and ii) the p.Gly136_Ser191 dup HELIOS variant on the protein level. Red inset = zinc finger 2 and 3 duplication. Var., variant; WT, wild-type; ZF, zinc finger; P1, patient 1

The index patient (P1) is a female born to non-consanguineous parents (Fig. 1A) who presented at birth with syndromic features of developmental abnormalities and immune dysregulation. Family history was unremarkable. She has profound bilateral sensorineural hearing loss, microcephaly, mild developmental delay, cleft palate, hypotonia, athelia, and dysmorphic facies (detailed in [12]). Consistent with underlying immune dysregulation, she has suffered from chronic anemia beginning at two months of age, which at times required hospitalization and red blood cell transfusions (Fig. 1B-F). She also had severe atopic dermatitis beginning at ten months of age affecting the face, arms, legs, and trunk. Her skin inflammation was initially refractory to optimal medical therapy (i.e. topical corticosteroids, emollients, bleach baths), but has become more manageable with age. The patient also experienced frequent upper respiratory tract infections, which reduced in frequency following initiation of monthly intravenous immunoglobulin (IVIG) replacement therapy (600 mg/kg every 4 weeks). Laboratory and hematologic evaluation revealed chronically positive direct antiglobulin tests (DAT) first detected at 9 weeks of age. Initially consistent with a mixed (cold > warm) AIHA phenotype (reduced haptoglobin, mildly increased bilirubin, and increased reticulocytes), which progressed to a predominantly warm AIHA phenotype (DAT positive) with age without overt hemolysis. The patient had two episodes of severe anemia requiring red blood cell transfusion. Other notable hematological features included a normocellular bone marrow with scattered hemophagocytic macrophages, no morphological evidence for dysplastic cells or a progressive marrow disorder, lymphopenia, increased ferritin, and elevated IgE (Table 1). Red cells did not carry any hemoglobin variants, although non-specific poikilocytosis and rouleaux formation were noted.

**Table 1:** Summary of major hematological and immunological laboratory parameters

	Patient (0.5–5 yo)	Reference (0.5–5 yo)
Lymphocytes (× 10^9^/L)	* 1.31	2.00–8.00
CD3^+^ T cells	1.14	0.90–4.50
CD4^+^ T cells	0.74	0.50–2.40
CD8^+^ T cells	* 0.19	0.30–1.60
CD19^+^ B cells	0.52	0.20–2.10
TREC (copy number/3µL)	* 3.08	> 75
Red blood cells (× 10^12^/L)	* 2.83	3.90–5.30
Reticulocytes (× 10^9^/L)	32	20–80
Nucleated red blood cells (× 10^9^/L)	* 0.05	< 0.01
Platelets (× 10^9^/L)	383	200–490
Mean platelet volume (fL)	9.9	8.60–11.6
Neutrophils (× 10^9^/L)	3.07	1.50–8.50
Monocytes (× 10^9^/L)	0.54	0.00–0.90
Hemoglobin (g/L)	* 103	105–135
Hematocrit	* 0.30	0.33–0.39
Mean corpuscular volume	* 104	75–87
Mean corpuscular hemoglobin	* 36	24–30
Prothrombin time (s)	9.9	9.6–11.9
International normalized ratio	0.9	0.9–1.1
Lactate dehydrogenase (U/L)	640	500–920
Antibody response		
IgG (g/L)	6.7^‡^	4.0–8.3
IgA (g/L)	0.63	0.18–1.00
IgM (g/L)	0.83	0.19–1.46
IgE (µg/L)	* 242	< 18
Tetanus antitoxin (IU/mL)	0.83	0.10–1.00
Diptheria antitoxin (IU/mL)	0.70	0.50–1.10
DAT (polyspecific)	*Positive + 3	Negative
Anti-IgG	*Positive + 3	Negative
Anti-IgA	Negative	Negative
Anti-IgM	Negative	Negative
Anti-C3c	Negative	Negative
Anti-C3d	Negative	Negative
Hemoglobinopathy workup		
HGB F	* 0.044	0.000–0.011
HGB A	* 0.831	0.848–0.920
HGB A2	0.029	0.022–0.034
BART’s or HGB H Spike	Not detected	Not detected

Tabulation of patient hematological and immunological laboratory values compared to age-specific reference ranges

*TREC* T cell receptor excision circle, *DAT* direct antiglobulin test, HGB, hemoglobin

^‡^value measured while patient was on intravenous immunoglobulin replacement

Given the unique constellation of features, P1 underwent trio whole genome sequencing (WGS) (detailed in [12]). This revealed a novel de novo germline heterozygous structural variant affecting *IKAROS zinc finger 2* (*IKZF2*) encoding HELIOS (NM_016260.3:c.406+540_574+13477dup; NP_057344.2:p.Gly136_Ser191dup) (Fig. 1G-H). The variant results in intron 5 at chr2:213900960 being joined to intron 6 chr2:213921017 (GRCh37) with a 2 bp GT insertion in between. This leads to tandem duplication of exon 5 (~ 20 kb duplication spanning exon 5 and parts of flanking introns), corresponding to tandem duplication of zinc fingers 2 and 3 of HELIOS (Fig. 1H). In our original description of ICHAD syndrome [12], we established that mRNA for the exon 5-duplicated *IKZF2* variant is expressed in patient primary cells.

### Characterization of the Impact of the p.Gly136_Ser191dup HELIOS Variant on Protein Expression and Localization

**Fig. 2:**
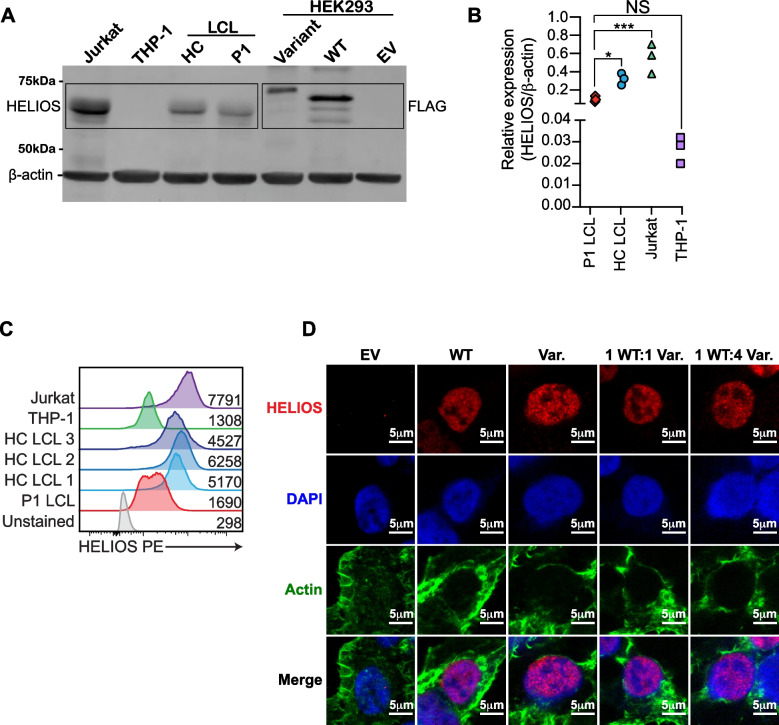
A novel *IKZF2* variant leads to reduced HELIOS protein expression and dominant interference of WT function. **A** HEK293 cells were transfected with empty vector (EV), wild-type (WT) *IKZF2*, or p.Gly136_Ser191dup (variant) *IKZF2*. Expression was determined by immunoblotting with both antibodies against both FLAG and HELIOS. *n* = 5. Expression of HELIOS was detected in patient-derived and control lymphoblastoid cell lines (LCLs) and compared to negative (THP-1 monocytic cells) and positive (Jurkat T leukemia cells) controls by **A**-**B**) immunoblot and **C**) flow cytometry. **D**) HEK293 cells were transfected with EV, WT, variant, or ratios of WT:variant and subjected to immunofluorescence for detection of HELIOS (red, Alexa Fluor 647), the nucleus (blue, DAPI), and F-actin (green, Phalloidin Alexa Fluor 488) using a Leica SP5 Confocal and LAS X Software with a 100 × objective lens. Scale bars = 5μm. *n* = 3. *p < 0.05, **p < 0.01, ***p < 0.001, ****p < 0.0001

The p.Gly136_Ser191dup HELIOS variant created a higher molecular weight HELIOS protein (as anticipated with a ~ 13 kDa 56 amino acid duplication) detected in transfected HEK293 cells (Fig. 2A and detailed in [12]). Total HELIOS protein expression was reduced overall in both transfected HEK293 (Fig. 2A) and patient-derived LCLs (Fig. 2A-C), however, cyclohexmide chase experiments indicated that the variant was stable when transfected into HEK293 cells (Fig. S1). As a TF, HELIOS must localize to the nucleus to regulate transcription [19]. To assess nuclear localization, we transfected HEK293 cells with wild type (WT) or p.Gly136_Ser191dup *IKZF2* alone, or increasing quantities of p.Gly136_Ser191 dup together with WT *IKZF2* (Fig. 2D) and we visualized protein expression by immunofluorescence. The variant protein was indistinguishable from WT protein in nuclear localization, nor did it affect the nuclear localization of WT protein. Despite this, as previously reported [12], the p.Gly136_Ser191dup *IKZF2* variant exhibits impaired normal repression of the *IL2* promoter both on its own and in a dominant negative and dose-dependent manner.

### Patient Lymphocyte Phenotyping Reveals NK Cell Lymphocytosis and CD3^+^ T Cell Lymphopenia, but Normal B Cell Frequencies

**Fig. 3:**
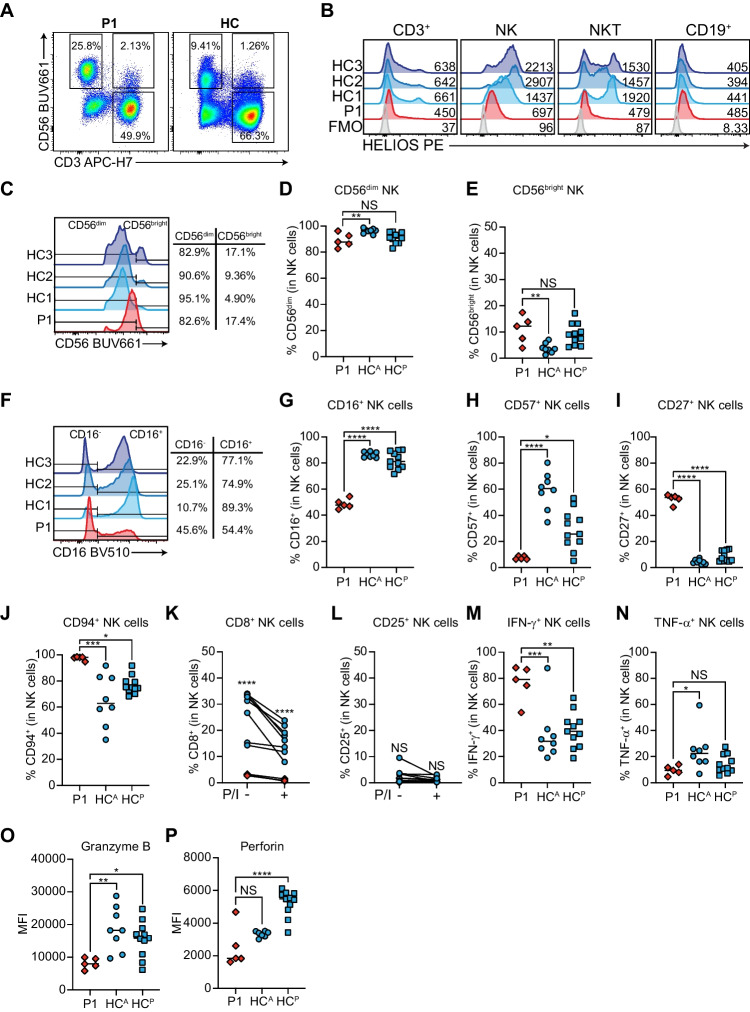
P1 has impaired NK cell development and function. **A** Representative flow cytometry dot plots showing CD3^+^CD56^−^ T cells, CD3^−^CD56^+^ NK cells, and CD3^+^CD56^+^ NKT cells for P1 and a control. **B** Representative HELIOS histograms for CD3^+^ T, NK, NKT, and CD19^+^ B cells in P1 and controls compared to a fluorescence minus one (FMO) control. Mean fluorescence intensities (MFI) are indicated. **C** Representative histograms of CD56 expression in P1 and control (HC1, HC2, HC3) CD3^−^CD56^+^ NK cells. Indicated is how CD56^dim^ and CD56^bright^ NK cells are defined as well as their associated frequencies. **D**-**E** Quantification of CD56^dim^ and CD56^bright^ frequencies. **F** Representative histograms of CD16 in P1 and control CD3^−^CD56^+^ NK cells. Indicated is how CD16^−^ and CD16^+^ NK cells are defined as well as their associated frequencies. Quantification of **G** CD16^+^, **H** CD57^+^, **I** CD27^+^, **J** CD94^+^ NK cells in P1 compared to adult (HC^A^) and pediatric (HC^P^) controls. **K**-**L** Frequency of **K** CD8^+^ and **L** CD25^+^ NK cells in patient and controls before and after 4 h PMA and ionomycin (P/I) stimulation. **M**–**N** Frequency of **M** IFN-γ^+^ and **N** TNF-α^+^ NK cells in P1 and controls after P/I stimulation. **O**-**P** MFI of **O** granzyme B and **P** perforin in unstimulated P1 and control NK cells. **A**-**J**, **M**-**P** P1 *n* = 5 independent blood draws, HC^A^ *n* = 8 individual controls, HC^P^ *n* = 11 individual controls. **K**-**L** P1 *n* = 3 independent blood draws, HC *n* = 8 individual controls. *p < 0.05, **p < 0.01, ***p < 0.001, ****p < 0.0001

HELIOS is highly expressed in CD3^+^ T cells and NK cells [18], both of which are known to be dysregulated in AIHA [4]. Furthermore, although Ikaros and Aiolos are both critical for B cell differentiation [38], the role of HELIOS in these cells is less clear. As such, we started by profiling the frequencies of CD3^+^ T, CD19^+^ B cells, CD3^−^CD56^+^ NK, and CD3^+^CD56^+^ NKT cells (Figs. 3A, S2A-D) and expression of HELIOS from P1 (*n* = 5 independent blood draws) and a cohort of adult (*n* = 8) and age-matched controls (*n* = 11). P1 had significantly reduced frequencies of CD3^+^ T (Fig. S2A), markedly elevated NK (Fig. S2B), and modestly elevated NKT (Fig. S2C) cells, all of which were HELIOS-deficient (Figs. 3B, S2E-G), while CD19^+^ B cell numbers were comparable to controls and showed normal HELIOS expression (Fig. S2D, H).

### P1 CD19^+^ B Cells Show Largely Normal Development but Increased TNF-α Production

HELIOS silencing is thought to maintain B cell function as ectopic expression leads to B cell hyperresponsiveness and lymphomagenesis [39]. We therefore studied the development and function of P1 B cells. In general, B cell development was largely normal (Fig. S3A), with intact naïve (Fig. S3B), switched memory [SM] (Fig. S3D), and plasmablasts (Fig. S3G-H). However, we did observe significantly increased non-switched memory [NSM] (Fig. S3C) and decreased transitional B cells (Fig. S3E-F), while HELIOS was lowly expressed in all B cell populations from both P1 and controls (Fig. S3I). In response to stimulation, patient B cells produced significantly more TNF-α than controls (Fig. S3J) across NSM (Fig. S3K), SM (Fig. S3L), and naïve (Fig. S3M) subsets.

### P1 NK Cells are Phenotypically Immature and Functionally Abnormal

Given the intriguing NK cell lymphocytosis, we set out to define the developmental and functional status of P1 NK cells by assessing the expression of CD56, maturation (CD27, CD57, CD94), adhesion (CD16), and activation (CD8, CD25) markers [40–42] on P1 NK cells compared to controls. The relative abundance of CD56^dim^ and CD56^bright^ NK cell populations was normal, although on histograms, the patient lacked a clear CD56^bright^ population (Fig. 3C-E). Strikingly, all maturation markers assessed were dysregulated in P1, including significantly reduced CD16^+^ (Fig. 3F-G**)** and CD57^+^ (Figs. 3H, S4A), but elevated CD27^+^ (Figs. 3I, S4B) and CD94^+^ (Figs. 3J, S4C) NK cells. Similarly, the frequency of CD8^+^ NK cells were significantly reduced in the patient both at baseline and in response to stimulation (Figs. 3K, S4D), while CD25^+^ NK cells (Figs. 3L, S4E) were normal. This abnormal distribution of markers disproportionately affected CD56^dim^ NK cells more than CD56^bright^ NK cells, although HELIOS was reduced in both populations (Fig. S4F-L). Taken together, patient NK cells are skewed towards an immature phenotype.

As NK cells mediate their effector functions through the release of proinflammatory cytokines and cytolysis [40], we studied whether these roles were affected in P1. In response to stimulation, P1 had significantly elevated IFN-γ^+^ NK cells (Figs. 3M, S4M), but comparable TNF-α^+^ NK cells (Figs. 3N, S4N). Similarly, P1 NK cells expressed significantly less granzyme B (Fig. 3O) and perforin (Fig. 3P), with this effect being more pronounced in CD56^dim^ NK cells (Fig. S4O-P). These defects were confirmed in multidimensional space when we clustered live CD3^−^CD56^+^ NK cells from P1 and adult and pediatric controls on CD16, CD27, CD57, CD94, HELIOS, perforin, granzyme B, IFN-γ, and TNF-α at baseline and in response to stimulation (Fig. S5).

### Patient CD8^+^ T Cells are Significantly Reduced, Have High PD-1 Expression, and are Potent Proinflammatory Cytokine Producers

**Fig. 4:**
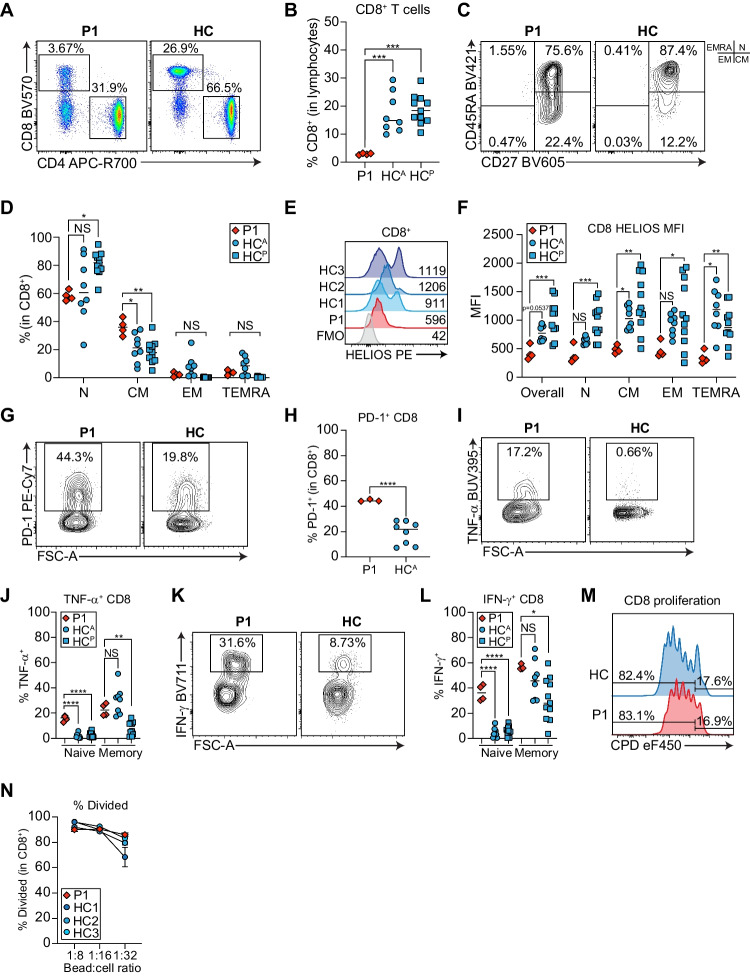
P1 CD8^+^ T cells are more differentiated and have enhanced cytokine production. **A** Representative dot plots showing CD3^+^CD4^−^CD8^+^ and CD3^+^CD4^+^CD8^−^ T cells. **B** Frequency of CD8^+^ T cells in P1 and adult (HC^A^) or pediatric (HC^P^) controls. **C** Representative contour plot of naïve (N), central memory (CM), effector memory (EM), and TEMRA CD8^+^ T cells in the patient and a control. Quadrants corresponding to each subset are shown to the right. **D** Quantification of naive, CM, EM, and TEMRA CD8^+^ T cells from **C**. **E** Representative HELIOS histograms for CD8^+^ T cells from P1 and three controls compared to a fluorescence minus one (FMO) control. Mean fluorescence intensities (MFI) are indicated. **F** Quantification of HELIOS MFI in different CD8^+^ T cell subsets. **G** Representative contour plots for PD-1^+^ CD8^+^ T cells in P1 and a control. **H** Quantification of **G**. **I**-**L** PBMCs stimulated 4 h with PMA + ionomycin. **I**, **K** Representative contour plots for TNF-α^+^ and IFN-γ^+^ CD8^+^ T cells in P1 and a control. **J**, **L** Quantification of TNF-α^+^ naïve and total memory CD8^+^ T cells and **L** IFN-γ^+^ naïve and total memory CD8^+^ T cells. **A**-**F**, **I**-**L** P1 *n* = 4 independent blood draws, HC^A^ *n* = 8 unique adult controls, HC^P^ *n* = 11 unique pediatric controls. **G**-**H** P1 *n* = 3 independent blood draws, HC^A^ *n* = 8 unique adult controls. *p < 0.05, **p < 0.01, ***p < 0.001, ****p < 0.0001. **M** Representative histograms for dilution of cell proliferation dye (CPD) eF450 in P1 or control CD8^+^ T cells after 4 days of stimulation with anti-CD3/CD28 beads at 1:32 bead to cell ratio. **N** Quantification of percent divided CD8^+^ T cells at 1:8, 1:16, 1:32 bead to cell ratios. Shown are technical duplicates

As CD8^+^ T cells are frequently enriched in autoimmune disease [43] and can be clonally expanded in AIHA [44], we assessed the frequency of CD8^+^ T cells and subsets and their HELIOS expression. P1 had profound CD8^+^ T cell lymphopenia (Fig. 4A-B), significantly elevated frequencies of central memory (CM) with a concurrent reduction in naïve subsets, while effector memory (EM) and TEMRA subsets were normal (Fig. 4C-D). Importantly, all P1 CD8^+^ T cell subsets were HELIOS-deficient (Fig. 4E-F).

PD- 1 is a critical inhibitory receptor that is upregulated in activated, memory, or exhausted T cells to restrain effector functions [45]. We therefore assessed PD-1 expression and proinflammatory cytokine production in P1 CD8^+^ T cells. In line with elevated CD8^+^ CM, we discovered significantly more PD-1 expression on P1 CD8^+^ T cells (Fig. 4G-H). These T cells are likely not exhausted as both naïve and total memory CD8^+^ T cells produced significantly more TNF-α (Fig. 4I-J) and IFN-γ (Fig. 4K-L) than controls, while CD8^+^ T cell proliferation was comparable to controls (Fig. 4M-N).

### P1 CD4^+^ T Cells are More Mature

**Fig. 5:**
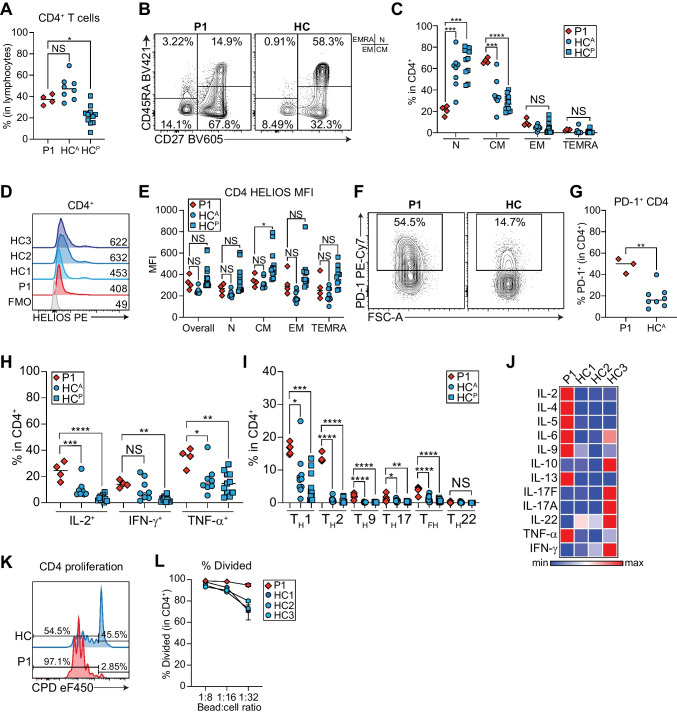
P1 CD4^+^ T cells are hyperactive and exhibit enhanced effector function. **A** Quantification of frequency of CD4^+^ T cells in P1 and adult (HC^A^) or pediatric (HC^P^) controls. **B** Representative contour plot of naïve (N), central memory (CM), effector memory (EM), and TEMRA CD4^+^ T cells in the patient and a control. Quadrants corresponding to each subset are shown to the right. **C** Quantification of naïve, CM, EM, and TEMRA CD4^+^ T cells from B). **D** Representative HELIOS histograms for CD4^+^ T cells in P1 and three controls compared to a fluorescence minus one (FMO) control. Mean fluorescence intensities (MFI) are indicated. **E** Quantification of HELIOS MFI in different CD4^+^ T cell subsets. **F** Representative contour plots for PD-1^+^ CD4^+^ T cells in P1 and a control. **G** Quantification of **F**. **H**-**I** PBMCs stimulated 4 h with PMA + ionomycin. **H** Quantification of IL-2^+^, IFN-γ^+^, and TNF-α^+^ CD4^+^ T cells in P1 and controls. **I** Quantification of IFN-γ^+^IL- 4^−^ T_H_1, IFN-γ^−^IL-4^+^ T_H_2, IL-21^+^IL-9^−^ T_FH_, IL-21^−^IL-9^+^ T_H_9 cells, IL-17^+^IL-22^−^ T_H_17, and IL-17^−^IL-22^+^ T_H_22 cells in P1 and controls. **A**-**C**, **H**-**I** P1 *n* = 4 independent blood draws, HC^A^ *n* = 8 unique adult controls, HC^P^ *n* = 11 unique pediatric controls, **D**-**E** P1 *n* = 3 independent blood draws, HC^A^ *n* = 8 unique adult controls. *p < 0.05, **p < 0.01, ***p < 0.001, ****p < 0.0001. **J**-**L** PBMCs stimulated with anti-CD3/CD28 beads at various bead to cell ratios for 4 days. **J** T_H_ cytokines measured by LEGENDplex. **K** Representative histograms for dilution of cell proliferation dye (CPD) eF450 in P1 or control CD4^+^ T cells stimulated at 1:32 bead to cell ratio. **L** Quantification of percent divided CD4^+^ T cells at 1:8, 1:16, 1:32 bead to cell ratios. Shown is the mean of technical duplicates

CD4^+^ T helper (T_H_) cells and their subsets are critical for both protective antimicrobial immunity and driving pathological states such as autoimmunity and atopic disease [46]. We therefore enumerated the frequency of total CD4^+^ T cells and subsets and HELIOS expression. P1 CD4^+^ T cells were modestly elevated (Fig. 5A) in line with CD8^+^ T lymphopenia. We also found similar subset distributions to P1 CD8^+^ T cells, including significantly increased CD4^+^ CM (Fig. 5B-C), an associated reduction in naïve, and normal EM and TEMRA subsets (Fig. 5C). HELIOS expression was low in all CD4^+^ subsets (Fig. 5D-E).

### P1 CD4^+^ T Cells are Hyperresponsive and Exhibit Effector Phenotypes

Treg-specific, but not complete, knockout of *Ikzf2* in mice leads to increased PD-1 expression on CD4^+^ T cells [27]. Surprisingly, P1 had three-fold higher PD-1^+^ CD4^+^ T cells than controls (Fig. 5F-G), which could signify higher activation status. We thus measured the frequency of T_H_ subsets and cytokine production. Mirroring our published *IL2* promoter luciferase data [12], we observed a striking increase in IL-2^+^ CD4^+^ T cells in P1 (Figs. 5H, S6A). This was not limited to IL-2, as the patient also had significantly increased IFN-γ^+^ and TNF-α^+^ CD4^+^ T cells (Figs. 5H, S6B-C) and most T_H_ subsets, including T_H_1, T_H_2, T_H_9, T_FH_, and T_H_17 (Figs. 5I, S6D-F). This also held true when we measured T_H_ cytokines in the supernatant of stimulated PBMCs, where we found markedly elevated concentrations of IL-2, IL-4, IL-5, IL-6, IL-9, IL-13, and TNF-α, even in response to lower bead:cell ratios (Figs. 5J, S6G-R). Surprisingly, we observed marked impairment in secreted IL-22 levels (Fig. S6P). In keeping with this picture of hyperactive CD4^+^ T cells, P1 CD4^+^ T cells more readily proliferated in response to stimulation than controls (97.1% divided in patient vs. 54.5% in control) (Fig. 5K-L).

### P1 has Normal Treg Frequencies and TSDR Methylation

**Fig. 6:**
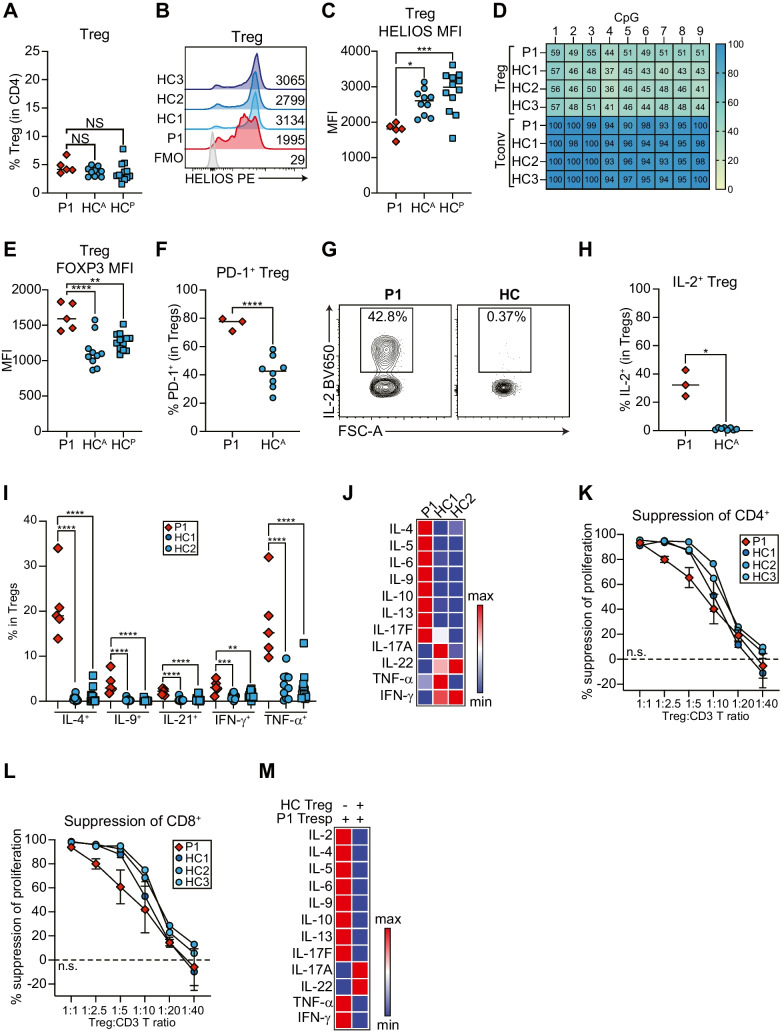
P1 Tregs have aberrant cytokine production and impaired suppressive function. **A** Quantification of frequency of CD3^+^CD4^+^CD8^−^CD25^+^CD127^lo^FOXP3^+^ Tregs in P1 and adult (HC^A^) or pediatric (HC^P^) controls. **B** Representative HELIOS histograms for Tregs in P1 and three controls compared to a fluorescence minus one (FMO) control. Mean fluorescence intensities (MFI) are indicated. **C** Quantification of HELIOS MFI in Tregs. **D** CpG methylation of the Treg-specific demethylated region (TSDR) for P1 or control Tregs and Tconvs. Percent methylation at different TSDR CpG sites is indicated. Representative of *n* = 2. **E** Quantification of FOXP3 MFI in Tregs. **F** Quantification of PD-1^+^ Tregs. **G**-**J** PBMCs stimulated 4 h with PMA + ionomycin. **G** Representative contour plot for IL-2^+^ Tregs. **H**-**I** Quantification of **H** IL-2^+^ and **I** IL-4^+^, IFN-γ^+^, IL-9^+^, IL-21^+^, and TNF-α^+^ Tregs. **A**-**C**, **E**, **I** P1 *n* = 5 independent blood draws, HC^A^ *n* = 10 unique adult controls, HC^P^ *n* = 12 unique pediatric controls. **F**–**H** P1 *n* = 3 independent blood draws, HC^A^ *n* = 8 unique adult controls. *p < 0.05, **p < 0.01, ***p < 0.001, ****p < 0.0001. **J** Isolated and expanded Tregs stimulated with anti-CD3/CD28 beads for 4 days. T_H_ cytokines measured by LEGENDplex. Shown are 3 technical replicates. **K**-**L** Isolated and expanded Tregs co-cultured at different ratios with control CD3^+^ T responder (Tresp) cells. *n* = 2 biological replicates. **K**-**L** Suppression of **K** CD4^+^ and **L** CD8^+^ T cell proliferation. Significance determined by one-way ANOVA of areas under the curve. **M** T_H_ cytokines measured by LEGENDplex when co-culturing 1:16 bead:cell ratio stimulated P1 CD3^+^ Tresp with control Tregs. Shown are technical triplicates

 ~ 70–80% of Tregs express Helios and at least in mice, this is thought to be important for regulating Treg identity and function [47]. As Tregs are critical mediators of tolerance/homeostasis and their dysfunction is associated with allergic and autoimmune disease [48], we enumerated their frequency and HELIOS expression in P1. P1 had normal frequencies of CD3^+^CD4^+^CD8^−^CD25^+^CD127^lo^FOXP3^+^ Tregs (Figs. 6A, S7A), but significantly reduced HELIOS expression (Fig. 6B-C).

Since methylation of the Treg-specific demethylated region (TSDR) in the *FOXP3* gene is important for maintaining stable FOXP3 expression and Treg suppressive function [49, 50], we quantified expanded P1 Treg TSDR methylation and FOXP3 expression in comparison to Tregs from healthy female controls. TSDR methylation was normal (Fig. 6D), although the average methylation trended to higher in P1 (Fig. S7B). Ex vivo P1 Tregs had significantly higher FOXP3 expression than controls, likely reflecting a higher state of activation **(**Fig. 6E).

### P1 Tregs are Predominantly PD-1^+^ and are Potent T_H_ Cytokine Producers

Given the striking increase in PD-1^+^ CD4^+^ (Fig. 5F-G) and CD8^+^ (Fig. 4G-H) T cells in P1, we assessed PD-1 expression in P1 ex vivo Tregs. Similarly, P1 had significantly more PD-1^+^ Tregs (~ 80%) than controls (~ 40%) (Figs. 6F, S7C). HELIOS genetic or pharmacological targeting in Tregs leads to elevated IL-2 [51], IFN-γ, and IL-17A [26, 27, 35, 52] and effector T_H_ cell signatures [53]. We thus studied T_H_ cytokine production in ex vivo Tregs and discovered significantly elevated IL-2^+^ Tregs in P1 (Fig. 6G-H), confirming our *IL2* luciferase data [12]. P1 also had a striking increase in IL-4^+^, IFN-γ^+^, IL-9^+^, IL-21^+^, and TNF-α^+^ Tregs (Figs. 6I, S7D-F). Expanded P1 Tregs also produced higher concentrations of IL-4, IL-5, IL-6, IL-9, IL-10, IL-13, and IL-17F (Fig. 6J, S7G-Q).

### P1 Tregs Have Impaired Suppressive Function and are not Intrinsically Resistant to Being Suppressed

The abnormal cytokine production in P1 Tregs prompted us to investigate their ability to suppress T responder (Tresp) proliferation and cytokine production. We cocultured expanded P1 and control Tregs at different ratios with stimulated control CD3^+^ Tresp. We discovered no significant differences between patient or control Treg-mediated suppression of CD4^+^ (Fig. 6K) or CD8^+^ (Fig. 6L) T cell proliferation, although suppression trended to lower in the 1:2.5 and 1:5 Treg:CD3 T cell ratios.

An intrinsic resistance to Treg-mediated suppression could also contribute to the patient’s prominent hyperactivation phenotype. We reversed our setup from above and cocultured control Tregs with P1 CD3^+^ Tresp and measured suppression of T_H_ cytokines. P1 T_H_ cytokine production was effectively suppressed by control Tregs (Figs. 6M, S8), excluding an intrinsic defect in susceptibility to suppression.

### scRNA-seq Reveals a Predominantly Memory and Effector Phenotype in P1 Tregs

**Fig. 7:**
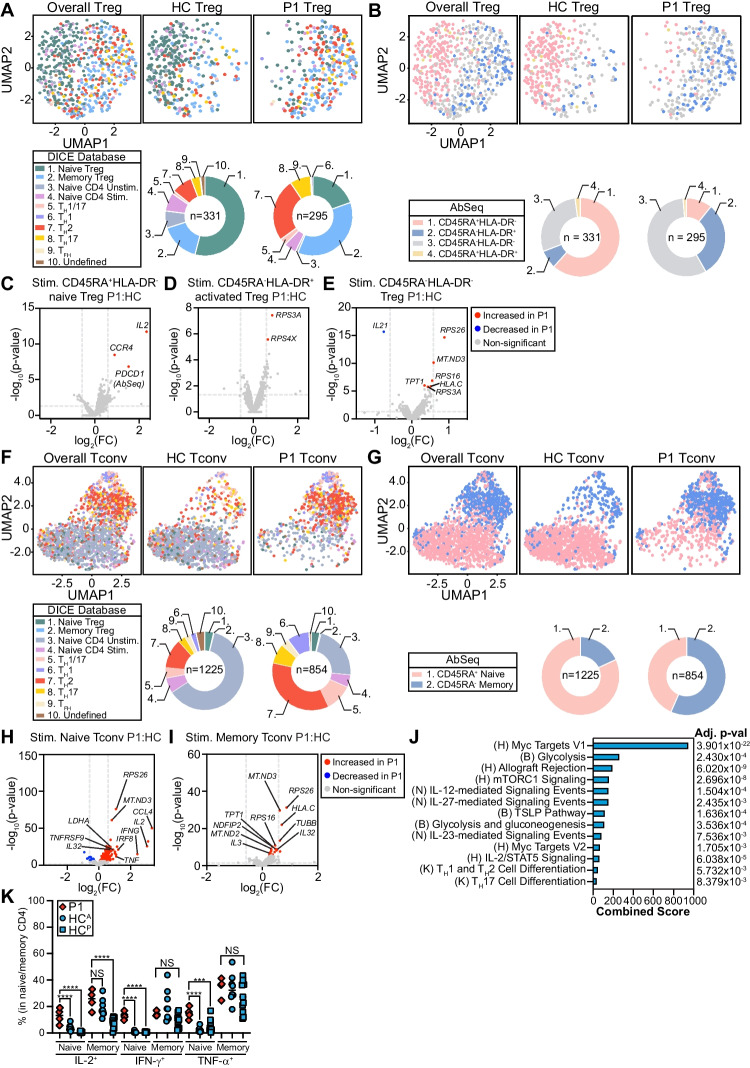
P1 naïve CD4^+^ T cells are more active and primed for effector function. **A**-**E** Single-cell RNA sequencing (scRNA-seq) of sorted CD3^+^CD4^+^CD25^+^CD127^lo^ Tregs from P1 and 2 age-matched and sex-matched controls. **A** Unstimulated Tregs clustered and annotated using the DICE project and **B** CD45RA/HLA-DR AbSeq labelling. Panels represent the controls and P1 Tregs clustered together (overall) or control or P1 Tregs alone. Included are legends with colours and numbers corresponding to each cell type labelled. Doughnut plots are included below each panel to represent the frequency of each annotated cell type. **C**-**E** Volcano plots comparing stimulated patient and control **C** CD45RA^+^HLA-DR^−^ naïve, **D** CD45RA^−^HLA-DR^+^ activated, and **E** CD45RA^−^HLA-DR^−^ Tregs. Red = significantly increased, blue = significantly decreased, gray = non-significant. Vertical dashed line: fold change = 1.5, horizontal dashed line: FDR = 0.05. Top 10 most significant genes are labelled. **F**-**I** scRNA-seq of sorted CD3^+^CD4^+^CD25^−^CD127^hi^ Tconv from P1 and 2 age-matched and sex-matched controls. **F**-**G** Unstimulated Tconv clustered and annotated as in **A-B**. **H**-**I** Volcano plots as in **C-E** comparing stimulated patient and control **H** CD45RA^+^ naïve or **I** CD45RA^−^ memory Tconvs. **J** Gene set enrichment of differentially expressed genes between stimulated naïve patient and control Tconvs using EnrichR. Shown are the combined scores and adjusted p-values. H = MSigDB Hallmark, B = BioPlanet 2019, *n* = NCI Nature 2016, K = KEGG 2021 Human. **K-P** PBMCs stimulated 4 h with PMA + ionomycin. Quantification of stimulated **K** IL-2^+^ naïve, IL-2^+^ total memory, IFN-γ^+^ naïve, IFN-γ^+^ total memory, TNF-α^+^ naïve, and TNF-α^+^ total memory CD4^+^ T cells. **K** P1 *n* = 4 independent blood draws, HC^A^ *n* = 8 unique adult controls, HC^P^ *n* = 11 unique pediatric controls. ***p < 0.001, ****p < 0.0001

To assess transcriptomic pathways that could drive the striking patient Treg phenotype, we sorted CD4^+^CD25^+^CD127^lo^ Tregs from P1 and two age-matched/sex-matched controls (Fig. S9A). We carried out whole transcriptome single-cell RNA sequencing (scRNA-seq) on Tregs from P1 (*n* = 295 cells) and controls (*n* = 331 cells) and performed dimensionality reduction using the uniform manifold approximation and projection (UMAP) method [54]. Patient Tregs clustered separately from controls (Fig. 7A) and when cell identity was annotated using the DICE project [55], they were predominantly labelled as memory Treg and T_H_ subsets (T_H_1 T_H_2, T_H_17, T_FH_), in line with the activated and aberrant cytokine phenotype observed (Fig. 6F-J). Similarly, annotation based on CD45RA AbSeq/HLA-DR AbSeq expression to identify CD45RA^+^HLA-DR^−^ naïve, CD45RA^−^HLA-DR^+^ activated, and CD45RA^−^HLA-DR^−^ Tregs [56, 57] also found that P1 Tregs were predominantly CD45RA^−^HLA-DR^+^ and CD45RA^−^HLA-DR^−^ (Fig. 7B). Nevertheless, P1 Treg subsets were transcriptomically similar to control Tregs (Figs. 7C-E, S9B-D), except for significantly increased *IL2*, *PDCD1*, and *CCR4* transcript abundance in P1 naïve Tregs (Fig. 7C), consistent with our flow cytometry data (Fig. 6G-H).

### scRNA-seq Reveals a Predominantly Memory and Effector Phenotype in P1 CD4^+^ Tconvs

Given the profound CD4 phenotype, we also sorted CD4^+^CD25^−^CD127^+^ conventional T cells (Tconv) cells from P1 and two age-matched/sex-matched controls and performed scRNA-seq (P1 *n* = 854 cells; control *n* = 1225 cells) (Fig. S9A). Like Tregs, P1 Tconv clustered separately from controls and were frequently labelled as T_H_ subsets (Fig. 7F) and CD45RA^−^ memory (Fig. 7G). When comparing the transcriptome of AbSeq-inferred naïve (Figs. 7H, S9E) and memory (Figs. 7I, S9F) CD4^+^ Tconv cells between P1 and controls, we found similar memory, but distinctly different naïve populations. Unstimulated naïve P1 CD4^+^ Tconv cells had significantly higher transcript abundance of genes related to IL-2 signalling, activation, and migration (*IL2RB*, *CXCR4*, *PDCD1*, *CCL5*, *JUN*). Stimulated P1 CD4^+^ Tconv cells had significantly higher expression of proinflammatory cytokine/chemokine genes (*IL2*, *IFNG*, *TNF*, *CCL4*), confirming our CD4^+^ phenotyping data (Fig. 5H).

### P1 CD4^+^ Tconvs are Poised for T_H_ Differentiation

To study what pathways could be driving naïve CD4^+^ Tconv differences, we carried out gene set enrichment analyses. Unstimulated naïve P1 CD4^+^ Tconv cells were more enriched in pathways related to proliferation, IL-6 signalling, allograft rejection, apoptosis, and hypoxia. Further, stimulated patient CD4^+^ Tconv cells showed significant enrichment in proliferation (Myc targets V1/V2), metabolism (glycolysis, gluconeogenesis, mTORC1 signaling), Treg survival (IL-2-STAT5), impaired tolerance (allograft rejection), atopy (TSLP pathway), and T_H_ differentiation (IL-27-/IL-12-/IL-23-mediated signalling events, T_H_1, T_H_2, T_H_17 cell differentiation) pathways (Fig. 7J). In line with the primed/poised phenotype of P1 naïve CD4^+^ Tconv cells, we observed significantly increased IL-2^+^, IFN-γ^+^, and TNF-α^+^ naïve P1 CD4^+^ T cells (Fig. 7K), with a similar trend observed in memory.

## Discussion

Germline pathogenic variants in *IKZF2* have only been discovered in humans in the past three years. The initial descriptions were of germline heterozygous or homozygous LOF *IKZF2* variants in a total of 13 patients with combined immunodeficiency and/or immune dysregulation [9–11] (summarized in Fig. S10, Table S3). Collectively, these germline LOF *IKZF2* variants were predominantly heterozygous (10/13) and were mostly associated with evidence of immune dysregulation (10/13) (Table S4), including systemic lupus erythematosus (SLE), hemophagocytic lymphohistiocytosis (HLH), idiopathic thrombocytopenic purpura (ITP), and Evan’s syndrome. Our group recently expanded this list through the discovery of 2 patients with germline DN *IKZF2* variants who presented with ICHAD syndrome [12]. Notably the patient carrying the DN pathogenic c.406+540_574+13477dup;p.Gly136_Ser191dup *IKZF2* variant (designated P1) experienced the most severe clinical manifestations of immune dysregulation out of all 15 patients described to date with pathogenic germline *IKZF2* variants. P1 presented with syndromic developmental features plus significant chronic AIHA and atopic dermatitis, features consistent with classification as a PIRD. Interestingly, their presentation showed some similarity to HLH [58], including elevated ferritin, impaired NK cell activity, scattered hemophagocytic macrophages in the bone marrow, and potentially increased soluble CD25. Given the profound immune dysregulation associated with the DN pathogenic c.406+540_574+13477dup;p.Gly136_Ser191dup *IKZF2* variant, we embarked on a detailed immunological assessment of P1.

Immunophenotyping of this patient with DN *IKZF2* deficiency revealed striking differences from previous homozygous and heterozygous LOF patients and mice. For example, P1 NK cells were phenotypically immature and likely hyperactive due to elevated IFN-γ production and reduced intracellular perforin and granzyme B expression (Fig. 3). In contrast, other reported patients had NK cell lymphopenia [10, 11] or normal NK development [9]. Furthermore, while *Ikzf2*^−/−^ mice have normal NK cell frequencies [28], NK cells from NKp46-mutant mice (Noé) show elevated Helios expression and effector function, which modulates protective memory CD4^+^/CD8^+^ T cell development [59]. The study of P1 has thus revealed a previously unappreciated role for HELIOS in human NK cell development and function but calls for more in-depth studies in more individuals in the future.

Helios has been extensively studied in mouse Tregs [47], but its role in human Tregs has remained relatively unclear. Previous CRISPR-mediated KO studies by our co-authors found that HELIOS is dispensable for lineage stability and suppressive function in fully differentiated Tregs [34], whereas germline homozygous or heterozygous LOF *IKZF2* variants caused a proinflammatory Treg phenotype associated with increased IL-2 and IFN-γ production, but intact suppressive function [9, 10]. Our functional studies with this DN *IKZF2* variant confirm that it is not required for Treg development, but that it is essential for inhibition of IL-2 expression and many other cytokines. Although functional suppression of T cell proliferation appeared normal, aberrant production of many T_H_ cytokines likely contributes to a functional defect in immune regulation in vivo (Fig. 6), which is exacerbated by the greater propensity of P1 naïve CD4^+^ T cells to differentiate into effector CD4^+^ T cell subsets (T_H_1, T_H_2, T_H_9, T_FH_). While striking, this observation will need to be confirmed in more affected individuals with DN *IKZF2* variants in the future since sample limitations prevented us from carrying out similar investigations on the other patient described previously [12].

Although *IKZF2* is highly homologous to and forms heterodimers with other members of the IKAROS family [47, 60], germline variants are associated with diverse clinical phenotypes. Specifically, *IKZF1* variants are associated with common variable immunodeficiency (CVID), cytopenias, malignancy, combined immunodeficiency (CID), and immune dysregulation [13, 14], DN and LOF variants in *IKZF3* are associated with B cell lymphopenia, malignancy, and susceptibility to *Pneumocystis jirovecii* pneumonia [15, 16], and LOF variants in *IKZF5* are associated with thrombocytopenia [17]. This diversity in phenotypes is likely explained by gene dosage effects, as shown with *IKZF1* variants [60], perturbing interactions with other IKAROS members. As is the case with *IKZF1* variants, it is possible that DN variants produce a more severe phenotype than that of loss of protein [61]. Furthermore, the more restricted cellular expression of certain IKAROS proteins (i.e. HELIOS) can also interact with these factors to influence clinical presentations. Future work focused on deciphering these intricate IKAROS family interactions will greatly improve our understanding of these disorders.

Taken together, we have defined the immune phenotype of a novel form of HELIOS deficiency caused by a DN variant, which leads to severe congenital AIHA and atopic dermatitis [12]. Major hallmarks include: largely normal B cells, NK cell lymphocyosis, immature NK cells, T cell lymphopenia (increased CD4^+^, reduced CD8^+^), low TRECs, and predominantly memory and effector CD4^+^ T cells and Tregs. Notably, the combination of CD4^+^ T cells poised to differentiate into T_H_ effector cells with aberrant NK cells likely contributes to the immune dysregulation. This study identifies new players in the pathogenesis of these immune-mediated disorders and has significantly expanded our understanding of human HELIOS, including its regulation of naïve CD4^+^ T cell differentiation into memory/effector subsets and NK cell development and function.

## Supplementary Information

Below is the link to the electronic supplementary material.

Supplementary file 1 (PDF 10173 KB)

## Data Availability

Sequence data that support the findings of this study have been deposited in Gene Expression Omnibus and can be accessed through the following reviewer token: mxmpqoakrpsxnqh.

## References

[1] Chan AY, Torgerson TR. Primary immune regulatory disorders: a growing universe of immune dysregulation. Curr Opin Allergy Clin Immunol. 2020;20(6):582–90. 32941318 10.1097/ACI.0000000000000689 PMC7769114

[2] Flinn AM, Gennery AR. Primary immune regulatory disorders: undiagnosed needles in the haystack? Orphanet J Rare Dis. 2022;17(1):99. 35241125 10.1186/s13023-022-02249-1 PMC8895571

[3] Seidel MG. Autoimmune and other cytopenias in primary immunodeficiencies: pathomechanisms, novel differential diagnoses, and treatment. Blood. 2014;124(15):2337–44. 25163701 10.1182/blood-2014-06-583260 PMC4192747

[4] Michalak SS, Olewicz-Gawlik A, Rupa-Matysek J, Wolny-Rokicka E, Nowakowska E, Gil L. Autoimmune hemolytic anemia: current knowledge and perspectives. Immun Ageing. 2020;17(1):38. 33292368 10.1186/s12979-020-00208-7 PMC7677104

[5] Zanella A, Barcellini W. Treatment of autoimmune hemolytic anemias. Haematologica. 2014;99(10):1547–54. 25271314 10.3324/haematol.2014.114561 PMC4181250

[6] Tranekaer S, Hansen DL, Frederiksen H. Epidemiology of secondary warm autoimmune haemolytic anaemia-a systematic review and meta-analysis. J Clin Med. 2021;10(6):1244. 33802848 10.3390/jcm10061244 PMC8002719

[7] Goldman FD, Gurel Z, Al-Zubeidi D, et al. Congenital pancytopenia and absence of B lymphocytes in a neonate with a mutation in the Ikaros gene. Pediatr Blood Cancer. 2012;58(4):591–7. 21548011 10.1002/pbc.23160 PMC3161153

[8] Kuehn HS, Boisson B, Cunningham-Rundles C, et al. Loss of B cells in patients with heterozygous mutations in IKAROS. N Engl J Med. 2016;374(11):1032–43. 26981933 10.1056/NEJMoa1512234 PMC4836293

[9] Hetemaki I, Kaustio M, Kinnunen M, et al. Loss-of-function mutation in IKZF2 leads to immunodeficiency with dysregulated germinal center reactions and reduction of MAIT cells. Sci Immunol. 2021;6(65):eabe3454. 34826260 10.1126/sciimmunol.abe3454

[10] Shahin T, Kuehn HS, Shoeb MR, et al. Germline biallelic mutation affecting the transcription factor Helios causes pleiotropic defects of immunity. Sci Immunol. 2021;6(65):eabe3981. 34826259 10.1126/sciimmunol.abe3981 PMC7612971

[11] Shahin T, Mayr D, Shoeb MR, et al. Identification of germline monoallelic mutations in IKZF2 in patients with immune dysregulation. Blood Adv. 2022;6(7):2444–51. 34920454 10.1182/bloodadvances.2021006367 PMC9006292

[12] Mohajeri A, Vaseghi-Shanjani M, Rosenfeld JA, et al. Dominant negative variants in IKZF2 cause ICHAD syndrome, a new disorder characterised by immunodysregulation, craniofacial anomalies, hearing impairment, athelia and developmental delay. J Med Genet. 2023;60(11):1092–104. 37316189 10.1136/jmg-2022-109127 PMC11206234

[13] Kuehn HS, Nunes-Santos CJ, Rosenzweig SD. IKAROS-associated diseases in 2020: genotypes, phenotypes, and outcomes in primary immune deficiency/inborn errors of immunity. J Clin Immunol. 2021;41(1):1–10. 33392855 10.1007/s10875-020-00936-x

[14] Hoshino A, Boutboul D, Zhang Y, et al. Gain-of-function IKZF1 variants in humans cause immune dysregulation associated with abnormal T/B cell late differentiation. Sci Immunol. 2022;7(69):eabi7160. 35333544 10.1126/sciimmunol.abi7160

[15] Yamashita M, Kuehn HS, Okuyama K, et al. A variant in human AIOLOS impairs adaptive immunity by interfering with IKAROS. Nat Immunol. 2021;22(7):893–903. 34155405 10.1038/s41590-021-00951-z PMC8958960

[16] Kuehn HS, Chang J, Yamashita M, et al. T and B cell abnormalities, pneumocystis pneumonia, and chronic lymphocytic leukemia associated with an AIOLOS defect in patients. J Exp Med. 2021;218(12). 10.1084/jem.20211118. 10.1084/jem.20211118 PMC8548914 34694366

[17] Lentaigne C, Greene D, Sivapalaratnam S, et al. Germline mutations in the transcription factor IKZF5 cause thrombocytopenia. Blood. 2019;134(23):2070–81. 31217188 10.1182/blood.2019000782

[18] Kelley CM, Ikeda T, Koipally J, et al. Helios, a novel dimerization partner of Ikaros expressed in the earliest hematopoietic progenitors. Curr Biol. 1998;8(9):508–15. 9560339 10.1016/s0960-9822(98)70202-7

[19] Hahm K, Cobb BS, McCarty AS, et al. Helios, a T cell-restricted Ikaros family member that quantitatively associates with Ikaros at centromeric heterochromatin. Genes Dev. 1998;12(6):782–96. 9512513 10.1101/gad.12.6.782 PMC316626

[20] Getnet D, Grosso JF, Goldberg MV, et al. A role for the transcription factor Helios in human CD4(+)CD25(+) regulatory T cells. Mol Immunol. 2010;47(7–8):1595–600. 20226531 10.1016/j.molimm.2010.02.001 PMC3060613

[21] Akimova T, Beier UH, Wang L, Levine MH, Hancock WW. Helios expression is a marker of T cell activation and proliferation. PLoS ONE. 2011;6(8):e24226. 21918685 10.1371/journal.pone.0024226 PMC3168881

[22] Serre K, Benezech C, Desanti G, et al. Helios is associated with CD4 T cells differentiating to T helper 2 and follicular helper T cells in vivo independently of Foxp3 expression. PLoS ONE. 2011;6(6):e20731. 21677778 10.1371/journal.pone.0020731 PMC3108993

[23] Leeansyah E, Svard J, Dias J, et al. Arming of MAIT cell cytolytic antimicrobial activity is induced by IL-7 and defective in HIV-1 infection. PLoS Pathog. 2015;11(8):e1005072. 26295709 10.1371/journal.ppat.1005072 PMC4546682

[24] Thornton AM, Korty PE, Tran DQ, et al. Expression of Helios, an Ikaros transcription factor family member, differentiates thymic-derived from peripherally induced Foxp3+ T regulatory cells. J Immunol. 2010;184(7):3433–41. 20181882 10.4049/jimmunol.0904028 PMC3725574

[25] Thornton AM, Lu J, Korty PE, et al. Helios(+) and Helios(-) Treg subpopulations are phenotypically and functionally distinct and express dissimilar TCR repertoires. Eur J Immunol. 2019;49(3):398–412. 30620397 10.1002/eji.201847935 PMC6402968

[26] Kim HJ, Barnitz RA, Kreslavsky T, et al. Stable inhibitory activity of regulatory T cells requires the transcription factor Helios. Science. 2015;350(6258):334–9. 26472910 10.1126/science.aad0616 PMC4627635

[27] Sebastian M, Lopez-Ocasio M, Metidji A, Rieder SA, Shevach EM, Thornton AM. Helios controls a limited subset of regulatory T cell functions. J Immunol. 2016;196(1):144–55. 26582951 10.4049/jimmunol.1501704 PMC4685018

[28] Cai Q, Dierich A, Oulad-Abdelghani M, Chan S, Kastner P. Helios deficiency has minimal impact on T cell development and function. J Immunol. 2009;183(4):2303–11. 19620299 10.4049/jimmunol.0901407

[29] Ross EM, Bourges D, Hogan TV, Gleeson PA, van Driel IR. Helios defines T cells being driven to tolerance in the periphery and thymus. Eur J Immunol. 2014;44(7):2048–58. 24740292 10.1002/eji.201343999

[30] Skadow M, Penna VR, Galant-Swafford J, Shevach EM, Thornton AM. Helios deficiency predisposes the differentiation of CD4(+)Foxp3(-) T cells into peripherally derived regulatory T cells. J Immunol. 2019;203(2):370–8. 31167776 10.4049/jimmunol.1900388 PMC6615958

[31] Himmel ME, MacDonald KG, Garcia RV, Steiner TS, Levings MK. Helios+ and Helios- cells coexist within the natural FOXP3+ T regulatory cell subset in humans. J Immunol. 2013;190(5):2001–8. 23359504 10.4049/jimmunol.1201379

[32] Gottschalk RA, Corse E, Allison JP. Expression of Helios in peripherally induced Foxp3+ regulatory T cells. J Immunol. 2012;188(3):976–80. 22198953 10.4049/jimmunol.1102964

[33] Ng MSF, Roth TL, Mendoza VF, Marson A, Burt TD. Helios enhances the preferential differentiation of human fetal CD4(+) naive T cells into regulatory T cells. Sci Immunol. 2019;4(41). 10.1126/sciimmunol.aav5947. 10.1126/sciimmunol.aav5947 PMC7340007 31757834

[34] Lam AJ, Uday P, Gillies JK, Levings MK. Helios is a marker, not a driver, of human Treg stability. Eur J Immunol. 2022;52(1):75–84. 34561855 10.1002/eji.202149318

[35] Nakagawa H, Sido JM, Reyes EE, Kiers V, Cantor H, Kim HJ. Instability of Helios-deficient Tregs is associated with conversion to a T-effector phenotype and enhanced antitumor immunity. Proc Natl Acad Sci U S A. 2016;113(22):6248–53. 27185917 10.1073/pnas.1604765113 PMC4896716

[36] Lu HY, Sharma M, Sharma AA, et al. Mechanistic understanding of the combined immunodeficiency in complete human CARD11 deficiency. J Allergy Clin Immunol. 2021;148(6):1559-1574 e1513. 33872653 10.1016/j.jaci.2021.04.006

[37] Sharma M, Fu MP, Lu HY, et al. Human complete NFAT1 deficiency causes a triad of joint contractures, osteochondromas, and B cell malignancy. Blood. 2022;140(17):1858–74. 35789258 10.1182/blood.2022015674

[38] Heizmann B, Kastner P, Chan S. The Ikaros family in lymphocyte development. Curr Opin Immunol. 2018;51:14–23. 29278858 10.1016/j.coi.2017.11.005

[39] Dovat S, Montecino-Rodriguez E, Schuman V, Teitell MA, Dorshkind K, Smale ST. Transgenic expression of Helios in B lineage cells alters B cell properties and promotes lymphomagenesis. J Immunol. 2005;175(6):3508–15. 16148093 10.4049/jimmunol.175.6.3508

[40] Abel AM, Yang C, Thakar MS, Malarkannan S. Natural killer cells: development, maturation, and clinical utilization. Front Immunol. 2018;9:1869. 30150991 10.3389/fimmu.2018.01869 PMC6099181

[41] Burk CM, Coffey KE, Mace EM, et al. Immunodeficiency, centromeric instability, and facial anomalies (ICF) syndrome with NK dysfunction and EBV-driven malignancy treated with stem cell transplantation. J Allergy Clin Immunol Pract. 2020;8(3):1103-1106 e1103. 31520839 10.1016/j.jaip.2019.08.040 PMC7064430

[42] Ruiz-Garcia R, Vargas-Hernandez A, Chinn IK, et al. Mutations in PI3K110delta cause impaired natural killer cell function partially rescued by rapamycin treatment. J Allergy Clin Immunol. 2018;142(2):605-617 e607. 29330011 10.1016/j.jaci.2017.11.042 PMC6109967

[43] Petrelli A, Mijnheer G, Hoytema van Konijnenburg DP, et al. PD-1+CD8+ T cells are clonally expanding effectors in human chronic inflammation. J Clin Invest. 2018;128(10):4669–4681. 10.1172/JCI96107 PMC6159979 30198907

[44] Smirnova SJ, Sidorova JV, Tsvetaeva NV, et al. Expansion of CD8+ cells in autoimmune hemolytic anemia. Autoimmunity. 2016;49(3):147–54. 26829107 10.3109/08916934.2016.1138219

[45] Kuchroo JR, Hafler DA, Sharpe AH, Lucca LE. The double-edged sword: Harnessing PD-1 blockade in tumor and autoimmunity. Sci Immunol. 2021;6(65):eabf4034. 34739340 10.1126/sciimmunol.abf4034 PMC13541067

[46] Zhu X, Zhu J. CD4 T helper cell subsets and related human immunological disorders. Int J Mol Sci. 2020;21(21):8011. 33126494 10.3390/ijms21218011 PMC7663252

[47] Thornton AM, Shevach EM. Helios: still behind the clouds. Immunology. 2019;158(3):161–70. 31517385 10.1111/imm.13115 PMC6797934

[48] Sakaguchi S, Mikami N, Wing JB, Tanaka A, Ichiyama K, Ohkura N. Regulatory T cells and human disease. Annu Rev Immunol. 2020;38:541–66. 32017635 10.1146/annurev-immunol-042718-041717

[49] Floess S, Freyer J, Siewert C, et al. Epigenetic control of the foxp3 locus in regulatory T cells. PLoS Biol. 2007;5(2):e38. 17298177 10.1371/journal.pbio.0050038 PMC1783672

[50] Polansky JK, Kretschmer K, Freyer J, et al. DNA methylation controls Foxp3 gene expression. Eur J Immunol. 2008;38(6):1654–63. 18493985 10.1002/eji.200838105

[51] Baine I, Basu S, Ames R, Sellers RS, Macian F. Helios induces epigenetic silencing of IL2 gene expression in regulatory T cells. J Immunol. 2013;190(3):1008–16. 23275607 10.4049/jimmunol.1200792 PMC3558938

[52] Wang ES, Verano AL, Nowak RP, et al. Acute pharmacological degradation of Helios destabilizes regulatory T cells. Nat Chem Biol. 2021;17(6):711–7. 34035522 10.1038/s41589-021-00802-w PMC8162940

[53] Yates K, Bi K, Haining WN, Cantor H, Kim HJ. Comparative transcriptome analysis reveals distinct genetic modules associated with Helios expression in intratumoral regulatory T cells. Proc Natl Acad Sci U S A. 2018;115(9):2162–7. 29440380 10.1073/pnas.1720447115 PMC5834721

[54] McInnes L, Healy J, Melville J. UMAP: uniform manifold approximation and projection for dimension reduction. 2018:arXiv:1802.03426.

[55] Schmiedel BJ, Singh D, Madrigal A, et al. Impact of genetic polymorphisms on human immune cell gene expression. Cell. 2018;175(6):1701-1715 e1716. 30449622 10.1016/j.cell.2018.10.022 PMC6289654

[56] Dong S, Maiella S, Xhaard A, et al. Multiparameter single-cell profiling of human CD4+FOXP3+ regulatory T-cell populations in homeostatic conditions and during graft-versus-host disease. Blood. 2013;122(10):1802–12. 23818545 10.1182/blood-2013-02-482539

[57] Rosenblum MD, Way SS, Abbas AK. Regulatory T cell memory. Nat Rev Immunol. 2016;16(2):90–101. 26688349 10.1038/nri.2015.1 PMC5113825

[58] Jordan MB, Allen CE, Greenberg J, et al. Challenges in the diagnosis of hemophagocytic lymphohistiocytosis: recommendations from the North American Consortium for Histiocytosis (NACHO). Pediatr Blood Cancer. 2019;66(11):e27929. 31339233 10.1002/pbc.27929 PMC7340087

[59] Narni-Mancinelli E, Jaeger BN, Bernat C, et al. Tuning of natural killer cell reactivity by NKp46 and Helios calibrates T cell responses. Science. 2012;335(6066):344–8. 22267813 10.1126/science.1215621

[60] Kuehn HS, Boast B, Rosenzweig SD. Inborn errors of human IKAROS: LOF and GOF variants associated with primary immunodeficiency. Clin Exp Immunol. 2023;212(2):129–36. 36433803 10.1093/cei/uxac109 PMC10128159

[61] Yamashita M, Morio T. Inborn errors of IKAROS and AIOLOS. Curr Opin Immunol. 2021;72:239–48. 34265590 10.1016/j.coi.2021.06.010

