# Supplementary material for: A Germline Heterozygous Dominant Negative IKZF2 Variant Causing Syndromic Primary Immune Regulatory Disorder and ICHAD
Source: J Clin Immunol. 2025 Apr 28;45(1):89. doi: 10.1007/s10875-025-01882-2 (PMC12037660; doi:10.1007/s10875-025-01882-2)
Supplement: Supplementary file 1 — (PDF 10173 KB) [file 10875_2025_1882_MOESM1_ESM.pdf]

SUPPLEMENTAL DATA

A germline heterozygous dominant negative *IKZF2* variant causing syndromic primary immune regulatory disorder and ICHAD

Henry Y. Lu[1,2], Maryam Vaseghi-Shanjani[1,2], Avery J. Lam[3,4], Mehul Sharma[1,2], Arezoo Mohajeri[5], Leandro B. R. Silva[1],Jana Gillies[3,4], Gui Xiang Yang[5], Susan Lin[1], Maggie P. Fu[5,6,7], Areesha Salman[5], Ronak Rahmanian[8], Linlea Armstrong[5], Jessica Halparin[1,9], Connie L. Yang[1,9], Mark Chilvers[1,10], Erika Henkelman[11], Wingfield Rehmus[1,12], Douglas Morrison[1,13], Audi Setiadi[1,13], Sara Mostafavi[5,14], Michael S. Kobor[5,7], Frederick K. Kozak[8], Catherine M. Biggs[1,15], Clara van Karnebeek[1,7], Kyla J. Hildebrand[1,15], Anna Lehman on behalf of the Care4Rare Canada Consortium[5], Megan K. Levings[3,4,16], Stuart E. Turvey[1,2,15]

[1]Department of Pediatrics, BC Children's Hospital, The University of British Columbia, Vancouver, BC, Canada
[2]Experimental Medicine Program, Faculty of Medicine, The University of British Columbia, Vancouver, BC, Canada
[3]BC Children's Hospital Research Institute, Vancouver, BC, Canada
[4]Department of Surgery, The University of British Columbia, Vancouver, BC, Canada
[5]Department of Medical Genetics, The University of British Columbia, Vancouver, BC, Canada
[6]Genome Science and Technology Program, Faculty of Science, The University of British Columbia, Vancouver, BC, Canada
[7]Centre for Molecular Medicine and Therapeutics, Vancouver, BC, Canada
[8]Division of Otolaryngology – Head & Neck Surgery, BC Children's Hospital, The University of British Columbia, Vancouver, BC, Canada
[9]Division of Hematology, Oncology & Bone Marrow Transplant, BC Children's Hospital, The University of British Columbia, Vancouver, BC, Canada
[10]Division of Respiratory Medicine, BC Children's Hospital, The University of British Columbia, Vancouver, BC, Canada
[11]Division of Plastic Surgery, BC Children's Hospital, The University of British Columbia, Vancouver, BC, Canada
[12]Division of Dermatology, BC Children's Hospital, The University of British Columbia, Vancouver, BC, Canada
[13]Department of Pathology and Laboratory Medicine, BC Children's Hospital, The University of British Columbia, Vancouver, BC, Canada
[14]Department of Statistics, The University of British Columbia, Vancouver, BC, Canada
[15]Division of Immunology, BC Children's Hospital, The University of British Columbia, Vancouver, BC, Canada
[16]School of Biomedical Engineering, The University of British Columbia, Vancouver, BC, Canada

Correspondence to: Stuart E. Turvey, MBBS, DPhil, FRCPC

BC Children's Hospital
950 West 28th Avenue
Vancouver, BC, V5Z 4H4, Canada
Phone: 604 875 2345 ext. 5094
Email: sturvey@bcchr.ca

SUPPLEMENTAL METHODS

Sanger sequencing

Genomic DNA was extracted from the whole blood of the patient and 2 healthy controls using a DNeasy Blood and Tissue Kit (Qiagen, Hilden, Germany). The breakpoint region was PCR amplified using GoTaq Green Master Mix (Promega, Madison, Wisconsin) using the following primers: Forward, 5'-GTACACTACACTACTCTGGA-3' and Reverse, 5'-GTGGTTGAGTATGTTTCCTAG-3' (Integrated DNA Technologies, Coralville, Iowa). The PCR product was purified using a QIAquick PCR Purification Kit (Qiagen) and sequenced using the same primers.

Plasmids were sequenced using the following primers:

Name	Sequence (5'-3')
Forward Primer 1	ACGCTTACAATTTCCATTCGCCA
Forward Primer 2	TGGCAGTACACCAATGGGCGTG
Forward Primer 3	GGCCATGATGAGGGTAGCAGCC
Forward Primer 4	GTCACCATGTACCTCCTATGGAAGA
Forward Primer 5	GCAGCCATGATGACCACCAGTCC
Forward Primer 6	GGAAGTTGCCACTCCAGTGCCC
Forward Primer 7	ACCGGTGGGACATTTGAGTTGCT
Forward Primer 8	TGTCATAGCTGTTTCCTGTGTGA
Forward Primer 9	TTTCCCCCTGGAAGCTCCCTCG
Forward Primer 10	TCCTTTGATCTTTTCTACGGGGT
Forward Primer 11	TGCTACAGGCATCGTGGTGTCA
Forward Primer 12	AGGGAATAAGGGCGACACGGAA
Forward Primer 13	CCAAACTGGAACAACACTCAACCC

HELIOS plasmid cloning

RNA was extracted from patient-derived and control LCLs using an RNeasy Plus Mini kit (#74034, Qiagen, Hilden, Germany) then converted to cDNA using the SuperScript III First-Strand Synthesis SuperMix (#11752050, ThermoFisher, Waltham, Massachusetts) according to manufacturer's recommendations. Restriction sites flanking *IKZF2* cDNA were introduced using KpnI/XbaI primers with Phusion Plus DNA Polymerase (#F630S, ThermoFisher) according to manufacturer's recommendations. The following primers were used: Forward, 5'-ATGCGGTACCGACGATGGAAACAGAGGCTATTGATG-3' (KpnI) and Reverse, 5'-GACTTCTAGACTAGTGGAATGTGTGCTCCC-3' (XbaI). PCR products were digested with KpnI and XbaI then cloned into a pCMV6-XL4-3xFLAG vector and confirmed by Sanger sequencing.

Transient plasmid transfections

For immunoblotting and immunofluorescence, HEK293 cells were seeded at $8.0x10^5$ cells/well in a 6-well plate in complete DMEM supplemented with 10% FBS (Gibco; Life Technologies, Carlsbad, California) and incubated for 24h at 37^0C. Cells wefre transfected with 3μg of plasmid DNA using the P3000, Lipofectamine 3000, and Opti-MEM Medium reagents according to manufacturer's recommendations. For immunofluorescence variant/WT ratios were varied by maintaining the total plasmid DNA amount the same (e.g. 1:1 p.Gly136_Val192dup/WT was 1.5μg variant + 1.5μg WT).

Cell culture and lymphoblastoid cell line (LCL) immortalization

PBMCs from the patient and controls were cultured or stimulated in complete RPMI-1640 (GE Healthcare) supplemented with 10% heat inactivated FBS (Gibco, Life Technologies, Rockville, Maryland), 2mM L-glutamine (HyClone; ThermoFisher Scientific, Waltham, Massachusetts), and 1mM sodium pyruvate (Gibco; Life Technologies, Carlsbad, California).

To generate Epstein-Barr virus (EBV)-transformed immortalized B cell lines, PBMCs from the patient and a healthy control were cultured in complete RPMI-1640 supplemented with tacrolimus (AG Scientific, San Diego, California) for 1h. Cells were then infected with

supernatant collected from the viral replication-permissive marmoset cell line B95-8 (ATCC, Manassas, Virginia) until sufficient B cell blasts were observed. LCLs were cultured in complete RPMI-1640 without tacrolimus.

Cycloheximide (CHX)

HEK293 cells were seeded and transfected as described above. After transfection, the cells were incubated at 37ºC for 48 hours to allow plasmid overexpression. Following incubation, cycloheximide (CHX) was added at a final concentration of 50µg/mL per well. Cells were then lysed at 0, 6, 12, and 24 hours post-CHX treatment, as previously described, and HELIOS stability was assessed by immunoblotting.

Immunoblotting

Whole cell lysates were prepared by lysing LCLs, THP-1, Jurkat T cells, and transfected HEK293s in a RIPA Lysis and Extraction Buffer (#89901, ThermoFisher) supplemented with Halt Protease and Phosphatase Inhibitor Cocktail (#78440, ThermoFisher). Lysates were separated by 10% SDS-PAGE, transferred onto polyvinylidene difluoride membranes (Immobilon-FL; MilliporeSigma, Billerica, Massachusetts), blocked with 5% bovine serum albumin (BSA) in Tris-buffered saline with Tween-20, incubated with primary antibodies for 18h at 4^0C, incubated with secondary antibodies for 1h at room temperature, and imaged on a LI-COR Odyssey infrared scanner (LI-COR Biosciences, Lincoln, Nebraska). The following primary antibodies were used: HELIOS (#89270S) and β-actin (#8457S, #3700S), all from Cell Signaling Technologies (Danvers, Massachusetts), and FLAG (clone M2, #F3165, Millipore Sigma). The secondary antibodies used were the following: goat anti-rabbit IgG Dylight 800 conjugated (#611-145-002-0.5, Rockland Immunochemicals, Pottstown, Pennsylvania) and goat anti-mouse IgG IRDye 680RD (#926-6870, LI-COR). Immunoblots were quantified using Image Studio Lite (v.5.2.5, LI-COR).

Immunofluorescence

12mm coverslips (Fisherbrand, Fisher Scientific, Waltham, Massachusetts) were sterilized with 70% ethanol and washed with PBS. Sterilized coverslips were placed in 6-well plates before HEK293 cells were seeded and transfected as described above. Cells

were subsequently fixed with 4% paraformaldehyde in PBS (#15710, Electron Microscopy Sciences, Hatfield, Pennsylvania) for 10min, washed with PBS, permeabilized with 0.01% Triton X-100 in PBS, and washed with PBS. Transfected cells were stained with HELIOs primary antibodies (#720419, Invitrogen, ThermoFisher), washed, stained with goat anti-rabbit IgG conjugated to Alexa Fluor 647 (#A32733, ThermoFisher) and Alexa Fluor 488 Phalloidin (#A12379, ThermoFisher), then washed. Coverslips were mounted in ProLong Gold Antifade Mountant with DAPI (#P36935, ThermoFisher) on Superfrost Plus Microscope Slides (Fisherbrand, Fisher Scientific). Slides were imaged on a Leica SP5 II laser Scanning Confocal Microscope (Leica Microsystems GmbH, Wetzlar, Germany). Images were analyzed using Fiji (NIH, Bethesda, Maryland).

Immunophenotyping and analysis

PBMCs from the patient or controls were thawed and stimulated with 50ng/mL of phorbol 12-myristate 13-acetate (PMA) and 1μM ionomycin (P/I) for 4h in the presence of GolgiStop (#554724, BD Biosciences, Franklin Lakes, New Jersey). Cells were stained with extracellular antibodies from panels 1-4 (**Supplemental Table 5**) then fixed and permeabilized using the eBioscience Foxp3 Transcription Factor Staining Buffer Set (#00-5523-00, Invitrogen, ThermoFisher) according to manufacturer's recommendations. Samples were subsequently stained with intracellular antibodies from panels 1-4 (**Supplemental Table 5**) and acquired on a FACSymphony flow cytometer (BD Biosciences). Compensation and voltages were determined according to single stain controls using species-specific Compensation Particles for antibodies (#552843, #552845, #552844, BD Biosciences) and ArC Amine Reactive Compensation Beads for viability dyes (#A10346, ThermoFisher). Gating, frequency, and expression analyses were carried out on FlowJo (BD Biosciences).

NK cell clustering analyses were carried out by gating on $CD3^-CD56^+$ NK cells from the patient and controls, exporting the NK cell populations, then concatenating the unstimulated and stimulated conditions from all individuals. The concatenated unstimulated and stimulated samples were clustered on CD16, CD27, CD57, CD94, IFN-γ, TNF-α, granzyme B, perforin, and HELIOS using the tSNE plugin, opt-SNE[1], 1000

iterations, perplexity at 30, learning rate at 18925, KNN algorithm set to exact (vantage point tree), and using the Barnes-Hut gradient algorithm.

Proliferation assays and analysis

Thawed PBMCs were seeded at 25,000, 50,000, or 100,000 cells/well in technical triplicate in 96-well plates and labelled with Cell Proliferation Dye eF450 (#65-0842-85, Invitrogen, ThermoFisher) for 10min at 37^0C in the dark. Cell labelling was stopped with cold complete RPMI-1640 for 5min at 4^0C, washed, then stimulated with 1:8, 1:16, 1:32 bead to PBMC ratios using anti-CD3/CD28-coated Dynabeads (#11141D, Human T-Expander, Gibco, ThermoFisher) for four days. Cells were stained with antibody panel 6 (**Supplemental Table 5**), acquired on a CytoFLEX (Beckman Coulter), and analyzed using FlowJo. Percent divided cells were quantified using the Proliferation Modelling plugin on FlowJo and peak fitting.

Treg and Tconv expansion

$CD4^+$ T cells were enriched from thawed patient or control PBMCs using a EasySep Human $CD4^+$ T Cell Enrichment Kit (#19052, Stemcell Technologies, Vancouver, BC). $CD4^+$ T cells were stained with panel 7 and sorted for $CD4^+CD25^+CD127^{lo}$ Tregs and $CD4^+CD25^-CD127^+$ Tconvs on an Astrios (Beckman Coulter, Brea, California) or FACS Aria (BD Bioscience) cell sorter. Tregs and Tconvs were plated on aAPCs, which are irradiated (75Gy) mouse L cells overexpressing hCD32, hCD58, hCD80 and loaded with 0.1μg/mL anti-CD3 (OKT3, University of British Columbia Antibody Lab, Vancouver, British Columbia). Tregs and Tconvs were cultured in complete X-VIVO 15 (#BE02-060Q, Lonza, Basel, Switzerland) supplemented with 5% human serum (#022-210, WISENT, St-Bruno, Quebec), 1% penicillin/streptomycin (Gibco, ThermoFisher), 2mM GlutaMAX (Gibco, ThermoFisher), and 15.97mg/L phenol red (Sigma-Aldrich, Burlington, Massachusetts). Tregs and Tconvs were expanded in X-VIVO 15 additionally supplemented with 1000 IU/mL IL-2 for Tregs and 100 IU/mL IL-2 for Tconvs (Proleukin, #02130181, Novartis, Basel, Switzerland) for seven days. IL-2 was refreshed every 2-3 days. Tregs and Tconvs were harvested on day 7 and rested in X-VIVO 15 supplemented with reduced IL-2 (100 IU/mL) for Tregs and no IL-2 for Tconvs. Tregs and Tconvs were

then allocated for T cell suppression assays, cytokine quantitation, and TSDR methylation.

T cell suppression assays

$CD3^+$ Tresp cells were enriched from thawed patient or control PBMCs using an EasySep Human T cell Isolation Kit (#17951, Stemcell Technologies). $CD3^+$ Tresp cells were labelled and stimulated as described in the 'Proliferation Assay' section and cocultured with expanded patient or control Tregs and Tconvs for four days. As positive control, $CD3^+$ Tresp cells were stimulated with beads alone. All $CD3^+$ Tresp cells were plated at a density of 30,000 cells/well. $CD4^+$ and $CD8^+$ T cell proliferation was calculated by determining the division index (DI) using proliferation modelling as described above. Percent suppression was calculated using the following formula: (1 – [DI of sample/DI of positive control]) x 100%.

Measuring Treg and Tconv cytokine production by LEGENDplex

Supernatant was collected from the following conditions: 1) expanded Treg and Tconv cells were seeded at 25,000 cells/well and stimulated with 100U/mL of IL-2 and a 1:1 anti-CD3/CD28-coated Dynabeads to cell ratio for four days; 2) co-cultured Tregs and Tresp from suppression assays; and 3) PBMCs stimulated for proliferation. Supernatants were harvested and analyzed using a LEGENDplex Human Th Cytokine Panel 12-plex (#741027, BioLegend) on a CytoFLEX (Beckman Coulter) and analyzed with Qognit software (BioLegend) according to manufacturer's recommendations.

TSDR methylation

Genomic DNA was isolated from expanded Tregs and Tconvs and bisulfite converted with an EZ DNA Methylation-Direct Kit (Zymo Research, Irvine, California). Samples were PCR amplified with the following primers: Forward, 5'-AGAAATTTGTGGGGTGGGGTAT-3' and Reverse, 5'-ATCTACATCTAAACCCTATTATCACAACC-3'. Pyrosequencing was performed using a PyroMark Q96 MD (Qiagen) using the following primer: 5'-AGAAATTTGTGGGGTGGG-3'.

Single-cell RNA sequencing of Tregs and Tconvs

Patient and age-matched and sex-matched control PBMCs were thawed and enriched for CD3$^+$ T cells using an EasySep Human T cell Isolation Kit (#17951, Stemcell Technologies). CD3$^+$ T cells were stained with panel 5 (**Supplemental Table 5**) and sorted for CD4$^+$CD25$^+$CD127^{lo} Tregs and CD4$^+$CD25$^-$CD127$^+$ Tconvs on a FACS Aria cell sorter (BD Biosciences) (**Supplemental Figure 7A**). Sorted cells were left unstimulated or stimulated with P/I for 4h. scRNA-seq sample preparation was performed following the BD Rhapsody Single Cell platform as previously described[2] using the Rhapsody Cartridge Reagent Kit (#633731), the Rhapsody Cartridge Kit (#633733), the Rhapsody cDNA Kit (#633733), the Human Single Cell Sample Multiplexing Kit (#633781), and the Whole Transcriptome Analysis (WTA) Amplification Kit (#633801) all from BD Biosciences. Briefly, cells from each sample were labelled with Sample Tags and CD279 (PD-1) AbSeq (#940015, clone EH12.1, barcode sequence: ATGGTAGTATCACGACGTAGTAGGGTAATTGGCAGT), CD45RA AbSeq (#940011, clone HI100, barcode sequence: AAGCGATTGCGAAGGGTTAGTCAGTACGTTATGTTG), and HLA-DR AbSeq (#940010, clone G46-6, barcode sequence: TGTTGGTTATTCGTTAGTGCATCCGTTTGGGCGTGG) Ab-Oligos (all from BD Biosciences) and pooled in cold sample buffer to obtain ~20,000 cells in 620mL for each of the unstimulated and stimulated samples. A nanowell cartridge was primed and loaded with pooled samples for 15min at room temperature using the Rhapsody Express instrument (#633702, BD Biosciences) then loaded with cell capture beads. The cartridge was washed, cells were lysed, beads were retrieved, and the sample underwent reverse transcription, exonuclease I treatment, and denaturation. WTA, AbSeq, and Sample Tag libraries were prepared using a combination of random priming and extension and a series of PCR steps. PCR products were purified using AMPure beads (#A63880, Beckman Coulter) and quality was checked using an Agilent DNA High Sensitivity Kit (#5067-4626, Agilent Technologies, Santa Clara, California) on a 2100 Bioanalyzer (Agilent Technologies). Libraries were pooled according to manufacturer's recommendations, diluted to 650pM with a 20% PhiX spike-in, and multiplexed for paired-end (2 x 115bp) sequencing on a NextSeq 2000 Sequencing System (Illumina) at 200 cycles P3 for 1.1 billion reads.

scRNA-seq bioinformatics analyses

FASTQ files were processed using the BD Rhapsody WTA Analysis Pipeline on SevenBridges (www.sevenbridges.com) according to manufacturer's recommendations, using the GRCh38 GENCODE v.29 reference genome and transcriptome annotation. Distribution-based error correction (DBEC)-adjusted molecule counts and the R package Seurat[3] was used for all downstream analyses as previously described[2]. Tregs and Tconvs were scaled and clustered separately. Cell identities were annotated with the SingleR package using fine labeling from the Database of Immune Cell Expression, Expression quantitative trait loci (eQTLs) and Epigenomics project (DICE)[4]. CD45RA AbSeq and/or HLA-DR AbSeq distributions were used to define different cell subsets, including $CD45RA^{-}HLA\text{-}DR^{+}$ Tregs, $CD45RA^{+}HLA\text{-}DR^{-}$ Tregs, $CD45RA^{-}HLA\text{-}DR^{-}$ Tregs, and $CD45RA^{+}$ naïve Tconvs and $CD45RA^{-}$ memory Tconvs. Differential gene expression analyses were accomplished using the Seurat negative binomial regression[5] as previously described[2]. Gene set enrichment was carried out using Enrichr[6] with genes that were significantly (FDR<0.05) different between comparisons.

REFERENCES

1. Belkina AC, Ciccolella CO, Anno R, Halpert R, Spidlen J, Snyder-Cappione JE. Automated optimized parameters for T-distributed stochastic neighbor embedding improve visualization and analysis of large datasets. *Nat Commun*. 2019;10(1):5415.
2. Sharma M, Fu MP, Lu HY, et al. Human complete NFAT1 deficiency causes a triad of joint contractures, osteochondromas, and B cell malignancy. *Blood*. 2022.
3. Butler A, Hoffman P, Smibert P, Papalexi E, Satija R. Integrating single-cell transcriptomic data across different conditions, technologies, and species. *Nat Biotechnol*. 2018;36(5):411-420.
4. Schmiedel BJ, Singh D, Madrigal A, et al. Impact of Genetic Polymorphisms on Human Immune Cell Gene Expression. *Cell*. 2018;175(6):1701-1715 e1716.
5. Hafemeister C, Satija R. Normalization and variance stabilization of single-cell RNA-seq data using regularized negative binomial regression. *Genome Biol*. 2019;20(1):296.
6. Chen EY, Tan CM, Kou Y, et al. Enrichr: interactive and collaborative HTML5 gene list enrichment analysis tool. *BMC Bioinformatics*. 2013;14:128.

SUPPLEMENTAL TABLE LEGENDS

Supplemental Table 1. Differentially expressed genes in Treg subsets. Genes that had an adjusted p-value<0.05 when comparing P1 to controls in unstimulated or stimulated $CD45RA^{+}HLA\text{-}DR^{-}$, $CD45RA^{-}HLA\text{-}DR^{+}$, $CD45RA^{-}HLA\text{-}DR^{-}$ Tregs. Shown are gene names, average log_2(fold change), percent reads in the patient or controls, and adjusted p-values.

Supplemental Table 2. Differentially expressed genes in Tconv subsets. Genes that had an adjusted p-value<0.05 when comparing P1 to controls in unstimulated or stimulated $CD45RA^{+}$ naïve or $CD45RA^{-}$ memory $CD4^{+}$ Tconvs. Shown are gene names, average log_2(fold change), percent reads in the patient or controls, and adjusted p-values.

Supplemental Table 3. Major clinical and laboratory features associated with pathogenic germline HELIOS variants. Tabulation of clinical and immunological features observed in all patients with pathogenic *IKZF2* variants described to date. Variants are ordered from the N-terminus to the C-terminus of HELIOS. Asterisks represent variants that have not been tested in the heterozygous state to assess whether they are dominant negative. Horizontal lines represent features that were not present. Het., heterozygous; LOF, loss-of-function; DN, dominant negative; ND, no data; HLH, hemophagocytic lymphohistiocytosis; AIHA, autoimmune hemolytic anemia; ITP, idiopathic thrombocytopenic purpura; SLE, systemic lupus erythematosus; EBV, Epstein-Barr virus; EM, effector memory; CM, central memory; Treg, regulatory T cells; NK, natural killer cells; IVIG, intravenous immunoglobulin replacement.

Supplemental Table 4. Red flags of germline HELIOS deficiency. Tabulation of the most frequently (>50% of patients) observed genetic, clinical, and laboratory features in patients.

Supplemental Table 5. Flow cytometry antibodies used. List of flow cytometry antibodies used to identify different cell subsets organized based on panel.

SUPPLEMENTAL FIGURE LEGENDS

Supplemental Figure 1. HELIOS variant protein stability. A) Representative immunoblot assessing WT and variant HELIOS protein stability over time in a cycloheximide chase experiment. B) Quantification of immunoblot data (n=3 experimental replicates).

Supplemental Figure 2. Lymphocyte frequencies and HELIOS expression. A) Quantification of A) $CD3^+$ T, B) $CD56^+$ NK, C) $CD3^+CD56^+$ NKT, and D) $CD19^+$ B cells. E-H) HELIOS mean fluorescence intensity (MFI) in A-D). A-C), E-G) P1 n=5 independent blood draws, HC^A n=8 unique adult controls, HC^P n=11 unique pediatric controls. D), H) P1 n=4 independent blood draws, HC^A n=8 unique adult controls, HC^P n=11 unique pediatric controls. *p<0.05, **p<0.01, ***p<0.001, ****p<0.0001.

Supplemental Figure 3. Patient B cell immunophenotyping. A) Representative contour plots for IgD^+CD27^- naïve (NB), IgD^+CD27^+ non-switched memory (NSM), and IgD^-CD27^+ switched memory (SM) B cells from P1 and a control. Quadrants corresponding to each subset are shown to the right. Frequencies are indicated. B-D) Quantification of A) for B) NB, C) NSM, and D) SM B cell frequencies. E) Representative contour plots for $IgM^{hi}CD38^{hi}$ transitional B cells (TrB) from P1 and a control. F) Quantification of E). G) Representative contour plots for $CD27^+CD38^+$ plasmablasts (PB). H) Quantification of G). I) Quantification of HELIOS mean fluorescence intensity (MFI) in different B cell subsets. J) Representative contour plots of TNF-α^+ B cells in P1 and a control. K-M) Quantification of TNF-α^+ K) NSM, L) SM, and M) NB cells in P1 and controls. P1 n=4 independent blood draws, HC^A n=8 unique adult controls, HC^P n=11 unique pediatric controls. *p<0.05, **p<0.01, ***p<0.001, ****p<0.0001.

Supplemental Figure 4. Extended NK cell phenotyping. A-B) Representative contour plots of A) $CD57^+$, B) $CD27^+$, C) $CD94^+$, D) $CD8^+$, and E) $CD25^+$ NK cells in P1 and a control. F-L) Quantification of F) $CD16^+$, G) $CD57^+$, H) $CD27^+$, I) $CD94^+$, J) $CD8^+$, K) $CD25^+$, L) HELIOS mean fluorescence intensity (MFI) in $CD56^{bright}$ and $CD56^{dim}$ NK cells. M-N) Representative contour plots of M) IFN-γ^+ and N) TNF-α^+ NK cells in P1 and a control. O-P) Quantification of O) granzyme B and P) perforin MFI from $CD56^{bright}$ and

CD56^{dim} NK cells. A-I), L-P) P1 n=5 independent blood draws, HC^{A} n=8 unique adult controls, HC^{P} n=11 unique pediatric controls. J-K) P1 n=3 independent blood draws, HC^{A} n=8 unique adult controls. *$p<0.05$, **$p<0.01$, ***$p<0.001$, ****$p<0.0001$.

Supplemental Figure 5. Multidimensional clustering of NK cells. A) Clustering of live NK cells unstimulated or stimulated with PMA+ionomycin. Colouring corresponds to P1 (red), HC^{A} adult controls (light blue), and HC^{P} pediatric controls (dark blue). B) Distribution of marker expression overlayed on clusters generated from A).

Supplemental Figure 6. Extended CD4^{+} T cell cytokine profiling. A-F) Representative contour plots for A) IL-2^{+}, B) IFN-γ^{+}, C) TNF-α^{+}, D) IFN-γ^{+}IL-4^{-} $T_{H}1$, IFN-γ^{-}IL-4^{+} $T_{H}2$, E) IL-21^{+}IL-9^{-} T_{FH}, IL-21^{-}IL-9^{+} $T_{H}9$ cells, and F) IL-17^{+}IL-22^{-} $T_{H}17$, and IL-17^{-}IL-22^{+} $T_{H}22$ cells in P1 and controls. Quadrants corresponding to each subset are indicated. Frequencies are included. G-R) C-N) PBMCs stimulated with anti-CD3/CD28 beads at 1:8, 1:16, and 1:32 bead to cell ratios for 4 days. Cytokine concentrations measured by LEGENDplex for G) IL-2, H) IL-4, I) IL-5, J) IL-6, K) IL-9, L) IL-10, M) IL-13, N) IL-17F, O) IL-17A, P) IL-22, Q) TNF-α, and R) IFN-γ. Error bars represent technical duplicates. A-F) P1 n=4 independent blood draws, HC^{A} n=8 unique adult controls, HC^{P} n=11 unique pediatric controls. *$p<0.05$, **$p<0.01$.

Supplemental Figure 7. Extended Treg phenotyping. A) Gating strategy for identifying CD3^{+}CD4^{+}CD25^{+}CD127^{lo}FOXP3^{+} Tregs. B) Average Treg-specific demethylation region (TSDR) methylation across CpG sites. n=2. C-F) Representative contour plots for C) PD-1^{+} and D) IL-4^{+} and IFN-γ^{+} Tregs, E) IL-9^{+} and IL-21^{+}, F) and TNF-α^{+} Tregs from P1 and a control. G-Q) Isolated and expanded Tregs or Tconv stimulated with anti-CD3/CD28 beads at 1:1 bead to cell ratio for 4 days. Cytokine concentrations were measured by LEGENDplex for G) IL-4, H) IL-5, I) IL-6, J) IL-9, K) IL-10, L) IL-13, M) IL-17A, N) IL-17F, O) IL-22, P) TNF-α, and Q) IFN-γ. Shown is the mean of technical triplicates.

Supplemental Figure 8. P1 Tresp cytokine production in presence of control Tregs. A-L) P1 CD3^{+} Tresp cells stimulated with anti-CD3/CD28 beads and cocultured with control Tregs. Cytokine concentrations were measured by LEGENDplex for A) IL-2, B) IL-4, C) IL-5, D) IL-6, E) IL-9, F) IL-10, G) IL-13, H) IL-17F, I) IL-17A, J) IL-22, K) TNF-α, and L) IFN-γ. Shown is the mean of technical triplicates.

Supplemental Figure 9. scRNA-seq workflow and extended analyses. A) General workflow for the preparation of samples for scRNA-seq. B-F) Volcano plots comparing unstimulated P1 and control B) CD45RA^{+}HLA-DR^{-} naïve Tregs, C) CD45RA^{-}HLA-DR^{+} activated Tregs, D) CD45RA^{-}HLA-DR^{-} Tregs, E) CD45RA^{+} naïve Tconvs, and F) CD45RA^{-} memory Tconvs. Red=significantly increased, blue=significantly decreased, gray=non-significant. Vertical dashed line: fold change=1.5, horizontal dashed line: FDR=0.05. Top 10 significant genes are labelled. G) Gene set enrichment of differentially expressed genes between unstimulated naïve patient and control Tconvs using EnrichR. Shown are the combined scores and adjusted p-values. H=MSigDB Hallmark, B=BioPlanet 2019, N=NCI Nature 2016, K=KEGG 2021 Human.

Supplemental Figure 10. Location and impact of human germline *IKZF2* variants. Schematic representation of the HELIOS protein and domains. Annotated are all germline *IKZF2* variants identified to date. The variant included in the current study is marked in red.

Figure S1

A.

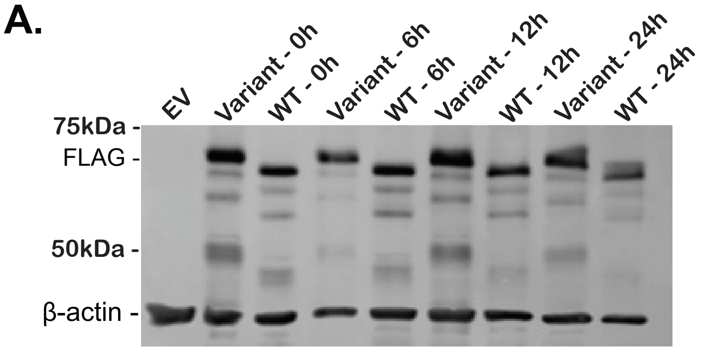
EV
Variant - 0h
WT - 0h
Variant - 6h
WT - 6h
Variant - 12h
WT - 12h
Variant - 24h
WT - 24h
75kDa -
FLAG -
50kDa -
β-actin -

B.

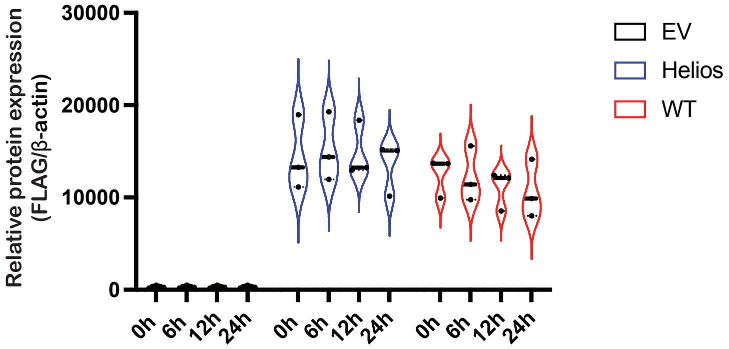
Relative protein expression (FLAG/β-actin)
30000
20000
10000
0
0h
6h
12h
24h
EV
Helios
WT

Figure S2

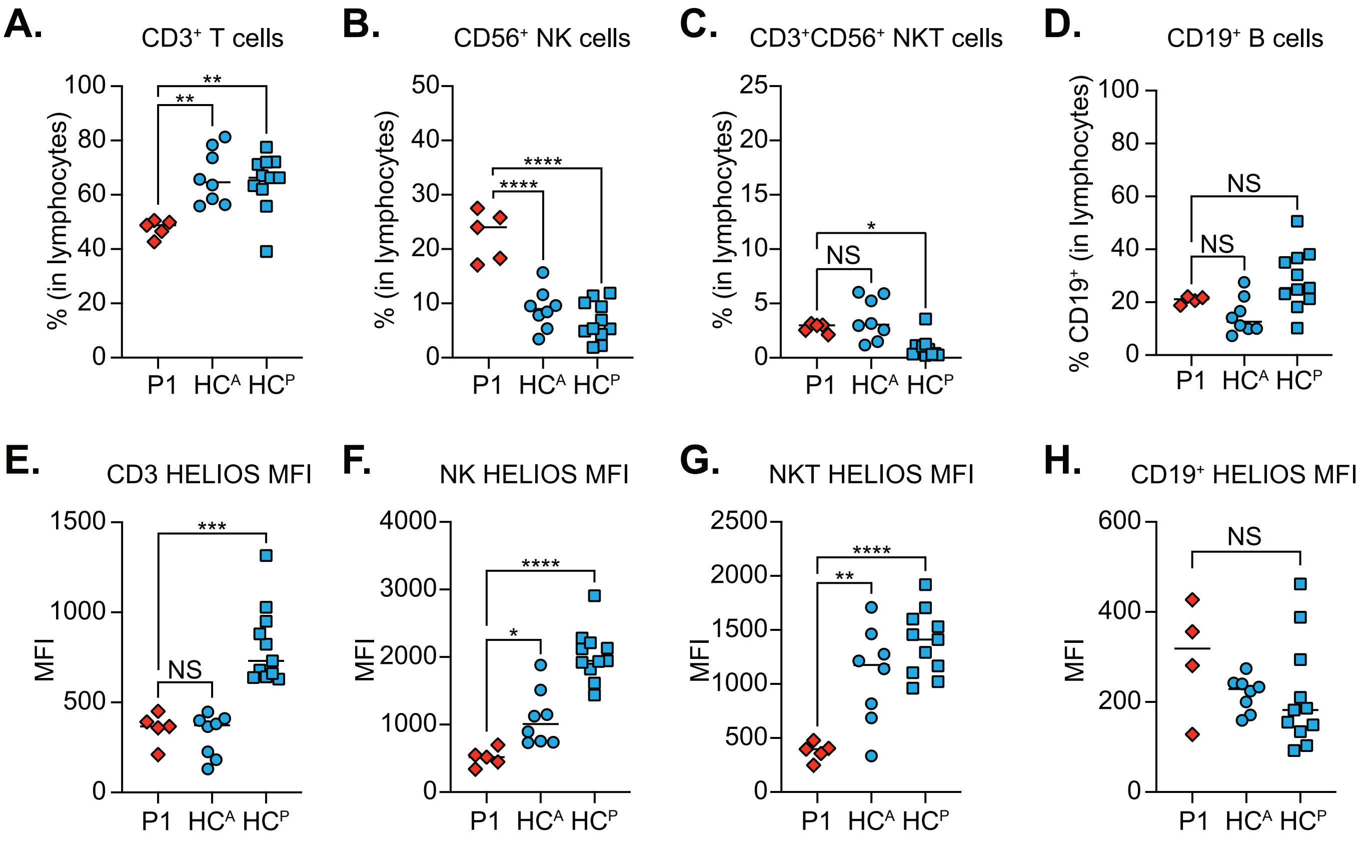
A.
CD3+ T cells
% (in lymphocytes)
100
80
60
40
20
0
**
**
P1 HCA HCP
B.
CD56+ NK cells
% (in lymphocytes)
50
40
30
20
10
0
****
****
P1 HCA HCP
C.
CD3+CD56+ NKT cells
% (in lymphocytes)
25
20
15
10
5
0
*
NS
P1 HCA HCP
D.
CD19+ B cells
% CD19+ (in lymphocytes)
100
80
60
40
20
0
NS
NS
P1 HCA HCP
E.
CD3 HELIOS MFI
MFI
1500
1000
500
0
***
NS
P1 HCA HCP
F.
NK HELIOS MFI
MFI
4000
3000
2000
1000
0
****
*
P1 HCA HCP
G.
NKT HELIOS MFI
MFI
2500
2000
1500
1000
500
0
****
**
P1 HCA HCP
H.
CD19+ HELIOS MFI
MFI
600
400
200
0
NS
P1 HCA HCP

Figure S3

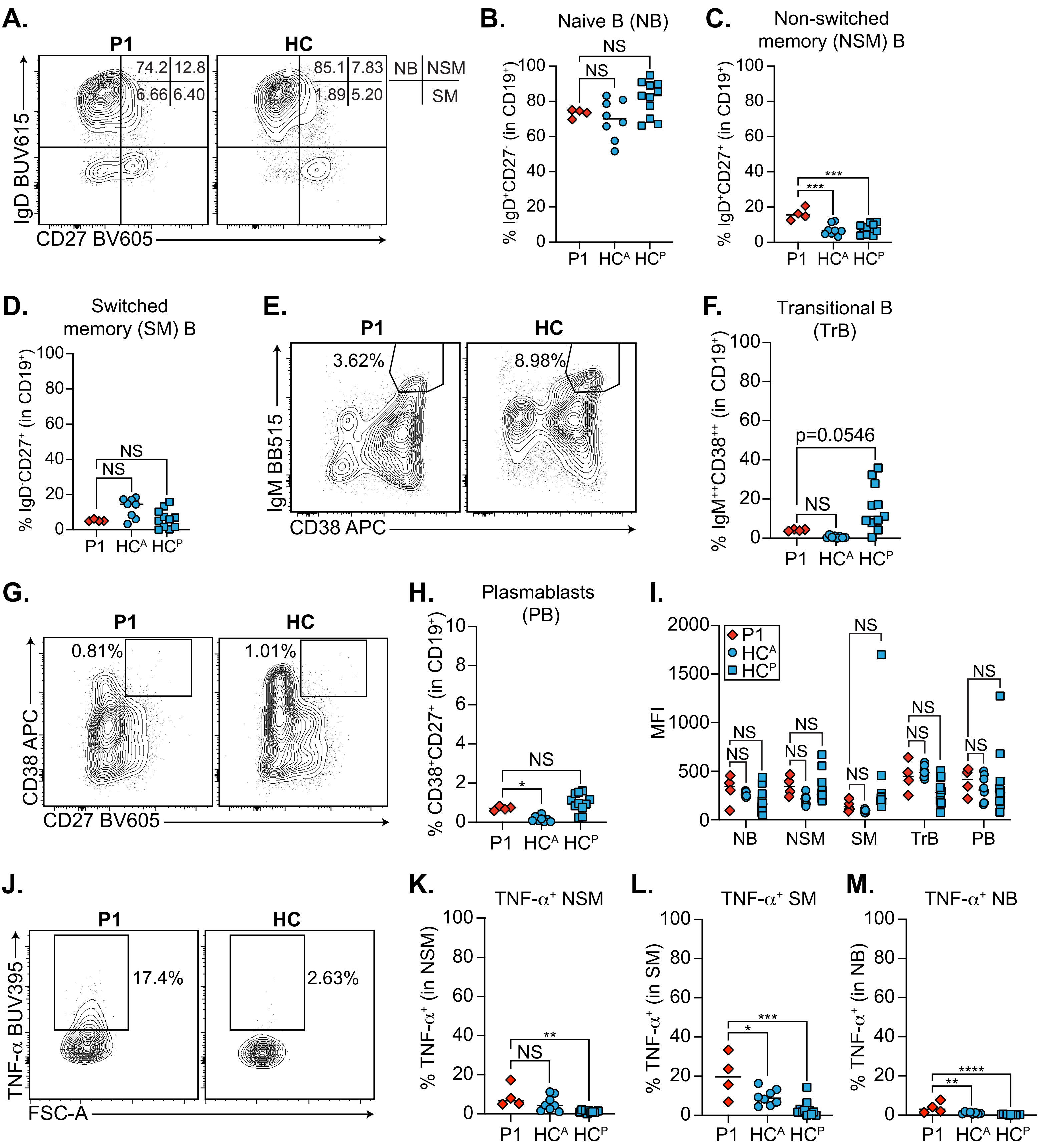
A.
P1
HC
74.2 12.8
6.66 6.40
85.1 7.83
1.89 5.20
NB NSM
SM
IgD BUV615
CD27 BV605
B.
Naive B (NB)
% IgD+CD27- (in CD19+)
NS
NS
P1 HCA HCP
C.
Non-switched memory (NSM) B
% IgD+CD27++ (in CD19+)
***
***
P1 HCA HCP
D.
Switched memory (SM) B
% IgD-CD27+ (in CD19+)
NS
NS
P1 HCA HCP
E.
P1
HC
3.62%
8.98%
IgM BB515
CD38 APC
F.
Transitional B (TrB)
% IgM++CD38++ (in CD19+)
p=0.0546
NS
P1 HCA HCP
G.
P1
HC
0.81%
1.01%
CD38 APC
CD27 BV605
H.
Plasmablasts (PB)
% CD38+CD27+ (in CD19+)
NS
*
P1 HCA HCP
I.
P1
HCA
HCP
MFI
NS
NB NSM SM TrB PB
J.
P1
HC
17.4%
2.63%
TNF-α BUV395
FSC-A
K.
TNF-α+ NSM
% TNF-α+ (in NSM)
**
NS
P1 HCA HCP
L.
TNF-α+ SM
% TNF-α+ (in SM)
***
*
P1 HCA HCP
M.
TNF-α+ NB
% TNF-α+ (in NB)
****
**
P1 HCA HCP

Figure S4

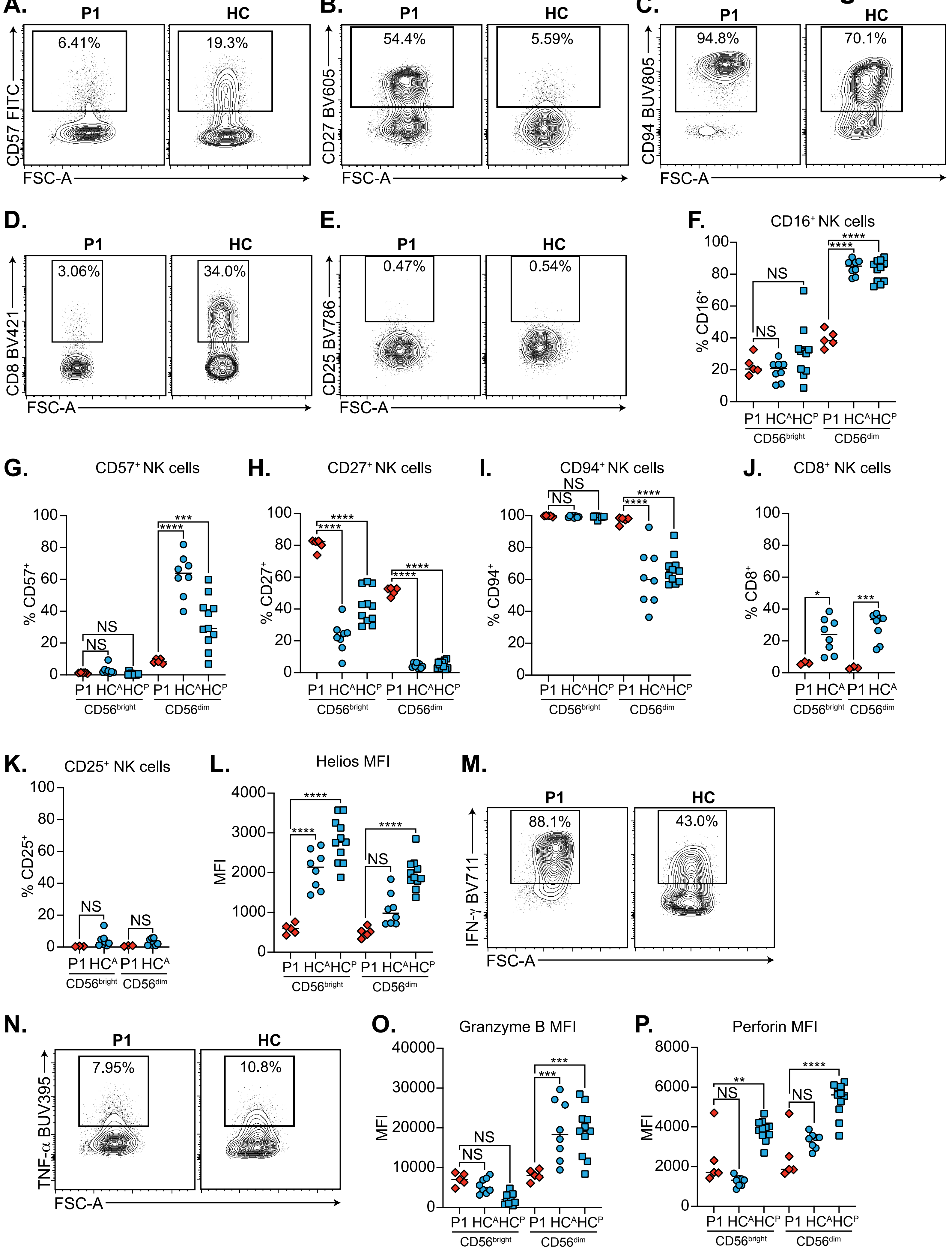
A.
P1
HC
6.41%
19.3%
CD57 FITC
FSC-A
B.
P1
HC
54.4%
5.59%
CD27 BV605
FSC-A
C.
P1
HC
94.8%
70.1%
CD94 BUV805
FSC-A
D.
P1
HC
3.06%
34.0%
CD8 BV421
FSC-A
E.
P1
HC
0.47%
0.54%
CD25 BV786
FSC-A
F.
CD16⁺ NK cells
% CD16⁺
CD56bright
CD56dim
G.
CD57⁺ NK cells
% CD57⁺
H.
CD27⁺ NK cells
% CD27⁺
I.
CD94⁺ NK cells
% CD94⁺
J.
CD8⁺ NK cells
% CD8⁺
K.
CD25⁺ NK cells
% CD25⁺
L.
Helios MFI
MFI
M.
P1
HC
88.1%
43.0%
IFN-γ BV711
FSC-A
N.
P1
HC
7.95%
10.8%
TNF-α BUV395
FSC-A
O.
Granzyme B MFI
MFI
P.
Perforin MFI
MFI

Figure S5

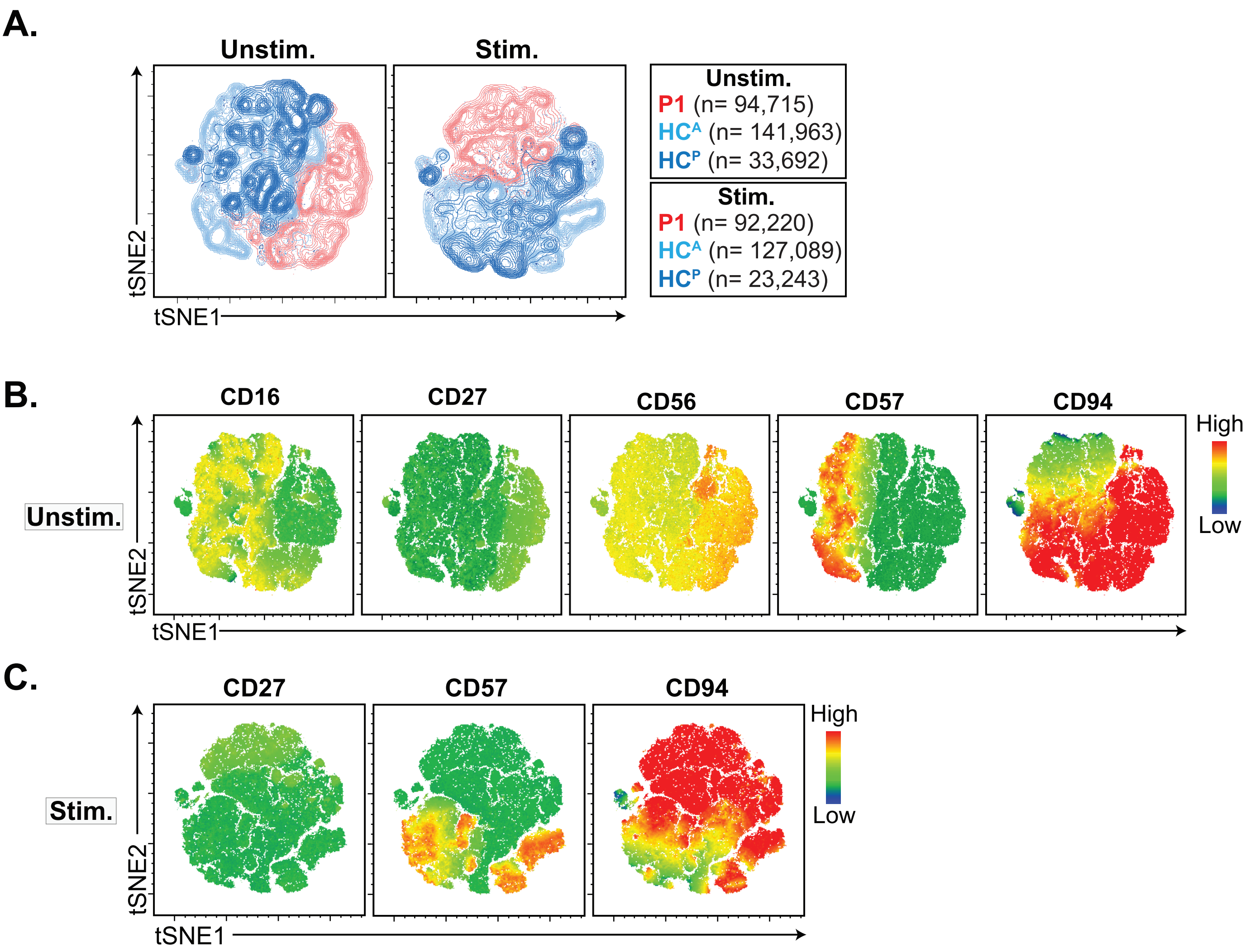
A.
Unstim.
Stim.
tSNE2
tSNE1
Unstim.
P1 (n= 94,715)
HCA (n= 141,963)
HCP (n= 33,692)
Stim.
P1 (n= 92,220)
HCA (n= 127,089)
HCP (n= 23,243)
B.
CD16
CD27
CD56
CD57
CD94
Unstim.
High
Low
C.
CD27
CD57
CD94
Stim.
High
Low

Figure S6

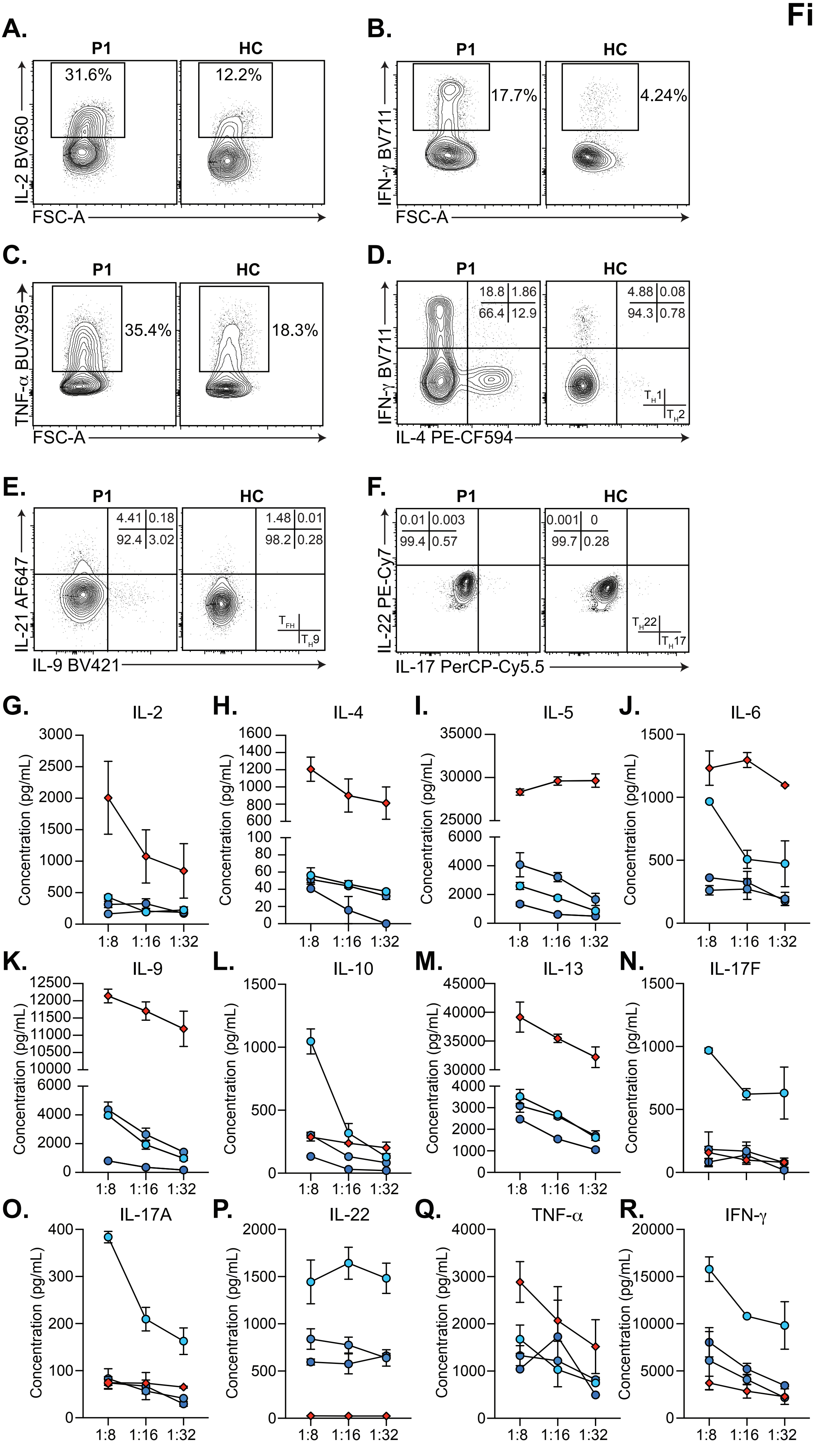
A.
P1
HC
31.6%
12.2%
IL-2 BV650
FSC-A
B.
17.7%
4.24%
IFN-γ BV711
C.
35.4%
18.3%
TNF-α BUV395
D.
18.8 1.86
66.4 12.9
4.88 0.08
94.3 0.78
IL-4 PE-CF594
E.
4.41 0.18
92.4 3.02
1.48 0.01
98.2 0.28
IL-21 AF647
IL-9 BV421
F.
0.01 0.003
99.4 0.57
0.001 0
99.7 0.28
IL-22 PE-Cy7
IL-17 PerCP-Cy5.5
G. IL-2
H. IL-4
I. IL-5
J. IL-6
K. IL-9
L. IL-10
M. IL-13
N. IL-17F
O. IL-17A
P. IL-22
Q. TNF-α
R. IFN-γ
Concentration (pg/mL)
1:8
1:16
1:32

Figure S7

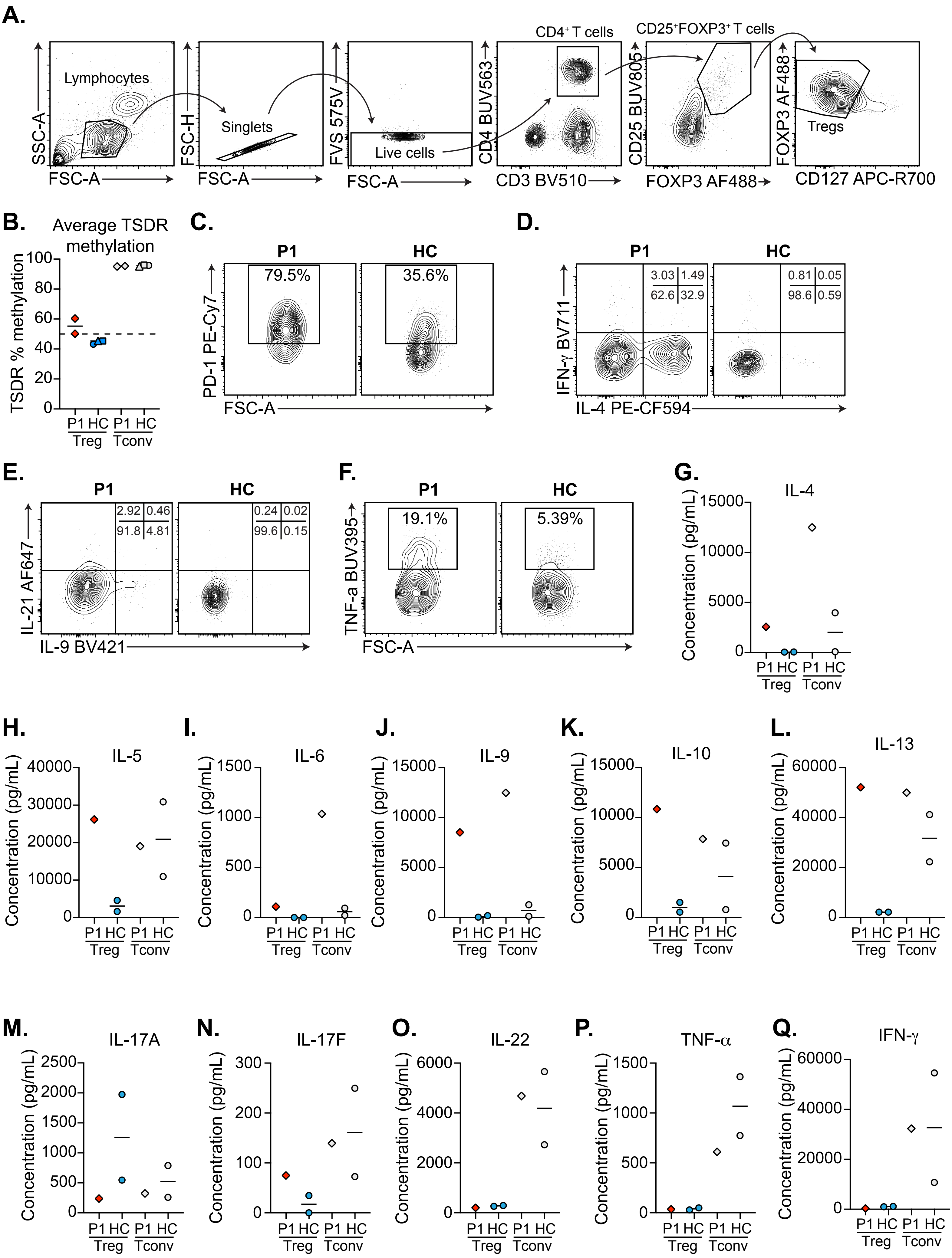
A.
Lymphocytes
Singlets
Live cells
CD4⁺ T cells
CD25⁺FOXP3⁺ T cells
Tregs
SSC-A
FSC-A
FSC-H
FVS 575V
CD4 BUV563
CD3 BV510
CD25 BUV805
FOXP3 AF488
CD127 APC-R700
B.
Average TSDR methylation
TSDR % methylation
P1 HC P1 HC
Treg Tconv
C.
P1
HC
79.5%
35.6%
PD-1 PE-Cy7
D.
3.03 1.49
62.6 32.9
0.81 0.05
98.6 0.59
IFN-γ BV711
IL-4 PE-CF594
E.
2.92 0.46
91.8 4.81
0.24 0.02
99.6 0.15
IL-21 AF647
IL-9 BV421
F.
19.1%
5.39%
TNF-a BUV395
G.
IL-4
Concentration (pg/mL)
H.
IL-5
I.
IL-6
J.
IL-9
K.
IL-10
L.
IL-13
M.
IL-17A
N.
IL-17F
O.
IL-22
P.
TNF-α
Q.
IFN-γ

Figure S8

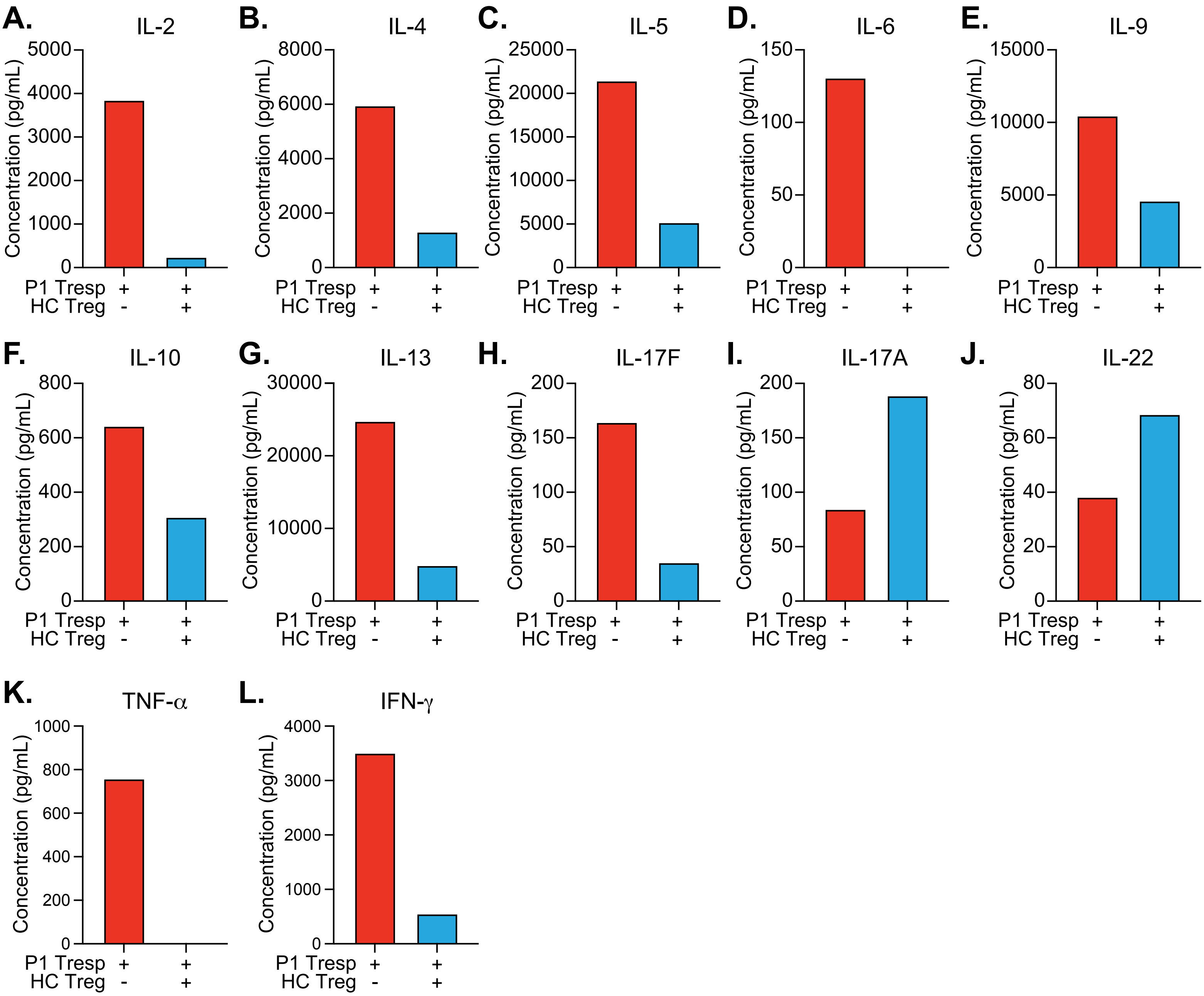
A.
IL-2
B.
IL-4
C.
IL-5
D.
IL-6
E.
IL-9
F.
IL-10
G.
IL-13
H.
IL-17F
I.
IL-17A
J.
IL-22
K.
TNF-α
L.
IFN-γ
Concentration (pg/mL)
P1 Tresp + +
HC Treg - +

Figure S9

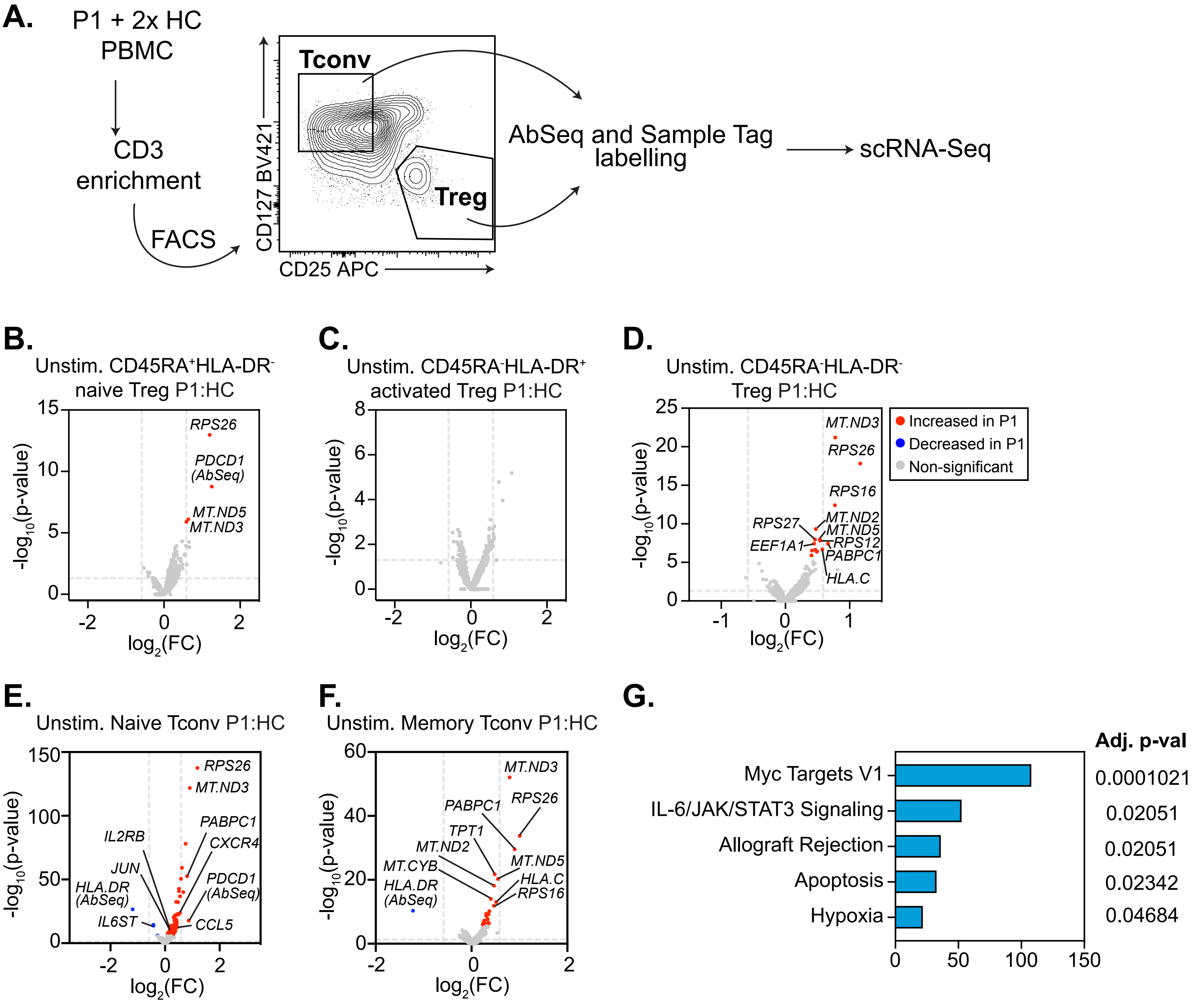
A.
P1 + 2x HC PBMC
CD3 enrichment
FACS
Tconv
Treg
CD127 BV421
CD25 APC
AbSeq and Sample Tag labelling
scRNA-Seq
B.
Unstim. CD45RA+HLA-DR- naive Treg P1:HC
RPS26
PDCD1 (AbSeq)
MT.ND5
MT.ND3
C.
Unstim. CD45RA-HLA-DR+ activated Treg P1:HC
D.
Unstim. CD45RA-HLA-DR- Treg P1:HC
MT.ND3
RPS26
RPS16
RPS27
MT.ND2
MT.ND5
EEF1A1
RPS12
PABPC1
HLA.C
Increased in P1
Decreased in P1
Non-significant
E.
Unstim. Naive Tconv P1:HC
RPS26
MT.ND3
PABPC1
CXCR4
IL2RB
JUN
HLA.DR (AbSeq)
PDCD1 (AbSeq)
IL6ST
CCL5
F.
Unstim. Memory Tconv P1:HC
MT.ND3
RPS26
PABPC1
TPT1
MT.ND2
MT.CYB
HLA.DR (AbSeq)
MT.ND5
HLA.C
RPS16
-log10(p-value)
log2(FC)
G.
Adj. p-val
Myc Targets V1
IL-6/JAK/STAT3 Signaling
Allograft Rejection
Apoptosis
Hypoxia
0.0001021
0.02051
0.02051
0.02342
0.04684

Figure S10

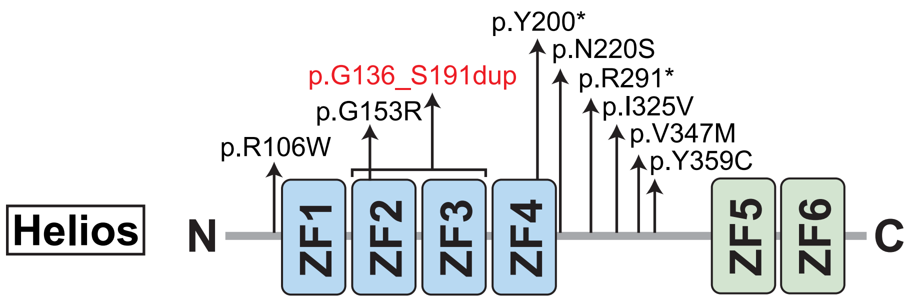
Helios
N
C
ZF1
ZF2
ZF3
ZF4
ZF5
ZF6
p.R106W
p.G153R
p.G136_S191dup
p.Y200*
p.N220S
p.R291*
p.I325V
p.V347M
p.Y359C

Table S1

Unstimulated CD45RA$^+$HLA-DR$^-$ Tregs P1/HC				
Gene	Average log$_2$(FC)	Patient % reads	Controls (n=2) % reads	Adjusted p-value
RPS26	1.20410776	0.781	0.275	2.05E-09
CD279 (PD-1) AbSeq	1.25536754	0.938	0.941	3.32E-05
MT.ND5	0.63759536	1	0.985	0.01554611
MT.ND3	0.58915204	1	1	0.02481357
**Stimulated CD45RA$^+$HLA-DR$^-$ Tregs P1/HC**				
Gene	Average log$_2$(FC)	Patient % reads	Controls (n=2) % reads	Adjusted p-value
IL2	2.33734243	0.304	0.188	3.65E-08
CCR4	0.89037551	0.478	0.062	6.64E-05
CD279 (PD-1) AbSeq	1.52169856	1	0.987	0.00287593
**Unstimulated CD45RA$^-$HLA-DR$^+$ Tregs P1/HC**				
Gene	Average log$_2$(FC)	Patient % reads	Controls (n=2) % reads	Adjusted p-value
—	—	—	—	—
**Stimulated CD45RA$^-$HLA-DR$^+$ Tregs P1/HC**				
Gene	Average log$_2$(FC)	Patient % reads	Controls (n=2) % reads	Adjusted p-value
RPS3A	0.85306912	0.979	0.846	0.00072689
RPS4X	0.6578455	0.957	0.872	0.05296446
**Unstimulated CD45RA$^-$HLA-DR$^-$ Tregs P1/HC**				
Gene	Average log$_2$(FC)	Patient % reads	Controls (n=2) % reads	Adjusted p-value
MT.ND3	0.77616704	0.994	1	1.24E-17
RPS26	1.16556698	0.754	0.253	2.90E-14
RPS16	0.77245575	0.928	0.714	7.73E-09
MT.ND2	0.4733298	1	1	9.40E-06
MT.ND5	0.53517757	0.988	1	0.00021311
RPS27	0.46032408	0.994	0.978	0.00021528
RPS12	0.54074794	0.988	0.956	0.00028853
PABPC1	0.66441632	0.94	0.802	0.00073903
EEF1A1	0.44542665	1	0.978	0.00073941
HLA.C	0.57526163	0.832	0.725	0.00421473
RPS27A	0.46500459	1	0.945	0.00463577
TPT1	0.41422608	1	0.989	0.00576331
RPS23	0.4942834	0.976	0.89	0.0073653
RPS13	0.48931195	0.982	0.912	0.00807184
MT.CYB	0.40709176	0.994	0.989	0.02477644
**Stimulated CD45RA$^-$HLA-DR$^-$ Tregs P1/HC**				
Gene	Average log$_2$(FC)	Patient % reads	Controls (n=2) % reads	Adjusted p-value

Table S1

IL21	-0.7570734	0.018	0.028	3.94E-12
RPS26	0.8831303	0.798	0.425	4.22E-11
MT.ND3	0.59101846	0.988	1	1.49E-06
RPS16	0.55000266	0.926	0.821	0.00265058
TPT1	0.34717656	1	1	0.0191753
HLA.C	0.51458739	0.859	0.708	0.02675433
RPS3A	0.43639648	1	0.991	0.03257976

Table S2

Unstimulated CD45RA$^+$ naïve Tconv P1/HC				
Gene	Average log_2(FC)	Patient % reads	Controls (n=2) % reads	Adjusted p-value
RPS26	1.18764709	0.835	0.251	4.85E-134
MT.ND3	0.9058343	1	0.998	3.14E-118
MT.ND5	0.75680139	0.995	0.994	1.54E-74
MT.ND2	0.61922654	0.997	1	1.16E-55
PABPC1	0.80699577	0.984	0.907	5.28E-49
MT.CYB	0.58768225	0.997	0.999	4.66E-47
MT.ND1	0.50909671	0.997	0.999	4.50E-39
MT.ATP6	0.51007248	1	0.999	3.25E-37
RPS16	0.66691202	0.938	0.78	1.53E-36
TPT1	0.54980831	1	0.996	3.06E-33
MT.CO1	0.40887775	1	1	6.28E-29
MT.CO3	0.47825594	1	1	1.10E-28
CD74 (HLA.DR) AbSeq	-1.1867976	0.989	0.99	5.39E-23
CXCR4	0.54563294	0.622	0.325	3.82E-20
RPS20	0.46388552	0.992	0.946	1.99E-19
EEF1A1	0.48001202	1	0.989	4.41E-19
HLA.C	0.4851569	0.93	0.803	7.04E-19
RPS3A	0.41255559	0.995	0.974	1.11E-18
RPS29	0.35973859	1	0.998	3.79E-18
PDE4DIP	0.3503569	0.281	0.079	2.72E-17
MT.ND4	0.31957801	0.997	1	1.13E-16
RPL37	0.40383317	0.997	0.961	5.64E-15
RPS27A	0.34302648	0.997	0.992	3.18E-14
CD279 (PD-1) AbSeq	0.86647915	0.93	0.924	3.78E-14
RPS3	0.42223693	0.884	0.733	1.07E-13
RPL10A	0.42589663	0.911	0.833	4.75E-13
RPS7	0.3963419	0.938	0.809	5.78E-13
RPS21	0.40979573	0.938	0.841	6.79E-13
RPS4X	0.4262625	0.986	0.922	9.16E-13
RPL3	0.4017918	0.938	0.878	3.85E-12
RPL5	0.38228455	0.959	0.896	1.41E-11
RPL34	0.3530331	1	0.989	1.48E-11
NAP1L1	0.42743514	0.884	0.798	1.96E-11
MT.ATP8	0.35716449	0.962	0.919	6.70E-11
RPL23A	0.34477365	0.989	0.935	6.74E-11
IL6ST	-0.4264239	0.203	0.432	7.26E-11
RPL37A	0.34709528	0.986	0.949	1.10E-10
MT.CO2	0.26563944	1	0.999	2.60E-10
LRRN3	-0.4252349	0.119	0.322	3.82E-10
RPS23	0.35711025	0.984	0.948	4.55E-10

Table S2

MIAT	0.19162171	0.135	0.015	7.17E-10
RPL6	0.35780755	0.976	0.911	1.09E-09
RPS27	0.31529748	0.997	0.988	2.12E-09
RPS8	0.35502653	0.992	0.975	2.69E-09
CCL5	0.45402733	0.122	0.005	5.41E-09
MT.ND4L	0.31499827	0.978	0.949	3.29E-08
IL2RB	0.17705987	0.111	0.012	4.25E-08
RTKN2	0.25091521	0.146	0.023	9.96E-08
EEF1B2	0.37647498	0.846	0.722	2.00E-07
RPL27	0.31837102	0.962	0.894	3.46E-07
AHNAK	0.28709213	0.257	0.095	3.85E-07
DHRS7	0.20209788	0.168	0.055	4.94E-07
RPL23	0.31184961	0.986	0.941	8.63E-07
RPS12	0.30558841	0.978	0.964	9.44E-07
RPL10	0.31995662	0.924	0.848	9.88E-07
ZNF90	0.27707266	0.438	0.28	1.08E-06
RPL39	0.29374602	0.995	0.967	1.48E-06
NHSL2	0.28545998	0.53	0.367	1.76E-06
EVI2B	0.33094594	0.543	0.351	1.78E-06
CENPK	0.1636345	0.135	0.036	3.39E-06
RPL38	0.31004347	0.935	0.88	3.53E-06
RPS6	0.28380205	1	0.991	4.14E-06
VIM	0.36951853	0.681	0.536	7.22E-06
JUN	0.21211469	0.173	0.067	7.94E-06
SYNE2	0.37496256	0.446	0.268	8.67E-06
RPL32	0.28319164	0.992	0.973	9.29E-06
02-Sep	0.27278652	0.386	0.241	9.73E-06
CSTB	0.24111641	0.3	0.155	1.17E-05
ARL4C	0.33146474	0.551	0.417	1.26E-05
JAZF1	0.19199078	0.159	0.047	2.62E-05
RPS15A	0.30448236	0.943	0.87	3.33E-05
TUBB	0.29222949	0.427	0.263	3.97E-05
RPL27A	0.35245909	0.873	0.791	4.32E-05
RPL13A	0.27241921	0.989	0.958	4.75E-05
EEF2	0.29929198	0.532	0.349	5.12E-05
SESN3	0.29666692	0.265	0.147	0.0001036
RPL35A	0.25816695	0.968	0.954	0.00013276
CD84	0.11816618	0.084	0.015	0.00013383
LINC01871	0.1132009	0.07	0.006	0.00018274
PLP2	0.20814347	0.184	0.068	0.00018419
RPL30	0.25554187	0.995	0.967	0.0002914
CD44	0.28085557	0.719	0.597	0.00041173
CCNI	0.29227033	0.695	0.565	0.00053983
ITGA4	0.21645487	0.208	0.091	0.00071131
IL32	0.25704165	0.368	0.218	0.00083061

Table S2

TAB2	0.23547289	0.351	0.193	0.00094034
RPL19	0.27438138	0.822	0.73	0.00094311
RPS5	0.29907769	0.808	0.721	0.00097342
ACTG1	0.31031392	0.77	0.668	0.00098564
CR1	0.15772106	0.111	0.028	0.00101276
RPLP2	0.27362006	0.868	0.785	0.00108883
SLC25A53	0.09662145	0.065	0.005	0.00124382
PABPC3	0.16096878	0.197	0.091	0.00159045
CD2	0.28822297	0.549	0.445	0.00169603
RPLP1	0.2575776	0.903	0.827	0.00248375
RPL21	0.22764356	0.995	0.967	0.00250351
MACF1	0.25404093	0.295	0.183	0.0026387
RPL41	0.22209607	0.995	0.978	0.00265406
RPS13	0.22008659	0.997	0.982	0.00283929
RPL9	0.22827667	0.986	0.973	0.00297188
SPOCK2	0.27135439	0.651	0.538	0.00335293
RPL13	0.27413158	0.816	0.697	0.00363511
ANXA2	0.10179614	0.07	0.014	0.0038377
CLDND1	0.18054461	0.146	0.064	0.00531297
PFDN5	0.25561479	0.592	0.435	0.00547469
RPL12	0.29663665	0.868	0.735	0.00748031
RPL11	0.23705899	0.973	0.953	0.00934594
MYL12B	0.24102862	0.573	0.466	0.01137859
LEF1	-0.2791661	0.503	0.608	0.01556475
RPL15	0.25246038	0.854	0.741	0.01680116
S100A4	0.14450502	0.111	0.041	0.01981441
RPL4	0.25523256	0.789	0.66	0.02561724
MYO9A	0.15146107	0.119	0.052	0.02658199
LDHA	0.20010635	0.332	0.214	0.02687985
SERPINB9	0.20540834	0.273	0.147	0.03192208
TBCD	0.08983582	0.086	0.025	0.03648794
ANXA1	0.22257962	0.259	0.147	0.0416569
RPL7	0.23760338	0.922	0.841	0.04625792
ZCCHC18	0.08348203	0.046	0.004	0.05331048
KLF6	0.21809127	0.281	0.177	0.0542021
**Stimulated CD45RA$^+$ naïve Tconv P1/HC**				
Gene	Average $\log_2$(FC)	Patient % reads	Controls (n=2) % reads	Adjusted p-value
RPS26	1.07510804	0.842	0.43	1.61E-72
MT.ND3	0.81265879	1	0.997	1.93E-57
CCL4	3.35267932	0.158	0.063	1.19E-46
RPS16	0.73852118	0.977	0.862	7.16E-31
CCL4L2	3.10429541	0.088	0.035	9.21E-29
B2M	0.47290926	1	0.999	2.63E-22
IL2	3.0415696	0.512	0.191	4.46E-22

Table S2

NDFIP2	0.62565985	0.591	0.272	5.81E-22
HSPA5	1.14864982	0.874	0.627	1.29E-21
LDHA	0.72694168	0.893	0.708	1.95E-21
RPL12	0.72767319	0.944	0.772	2.99E-20
HLA.C	0.61961593	0.893	0.74	3.44E-20
MT.ND5	0.51132604	0.991	0.976	5.79E-20
RPS23	0.51825583	1	0.966	2.09E-18
IL32	0.70567663	0.563	0.212	3.79E-18
IRF8	1.21888756	0.54	0.207	1.36E-17
TNFRSF9	0.9033837	0.4	0.07	1.54E-17
PTMA	0.4933372	1	0.995	1.79E-17
RAN	0.58851994	0.916	0.819	1.94E-17
HSP90AB1	0.53639726	1	0.987	1.99E-17
ODC1	0.63714795	0.693	0.407	7.59E-17
MT.CYB	0.44035863	0.995	0.997	1.59E-16
MT.ND2	0.40356237	1	1	2.12E-16
BCL2L11	0.60639601	0.363	0.108	2.66E-16
NPM1	0.53444938	0.995	0.984	2.97E-16
MIR155HG	0.77993904	0.981	0.904	1.30E-15
CD2	0.70941712	0.898	0.671	3.16E-15
FABP5	1.01295629	0.623	0.244	1.33E-14
CD48	0.65118101	0.693	0.457	3.49E-14
RPS6	0.46795979	1	0.993	3.83E-14
EGR1	-0.9263666	0.549	0.732	4.32E-14
MT.CO1	0.44274492	1	1	4.53E-14
PGAM1	0.62518598	0.581	0.278	8.34E-14
PDIA6	0.57175931	0.516	0.219	9.53E-14
TPT1	0.42309298	1	1	1.07E-13
RPL10A	0.46714299	0.995	0.941	1.26E-13
TUBA1B	0.5406853	0.665	0.403	2.76E-13
RPL3	0.4888052	0.963	0.899	5.13E-13
RPL37	0.46649178	0.986	0.974	8.00E-13
HSPA8	0.6295723	0.823	0.627	1.12E-12
RPS29	0.39926115	1	0.999	1.30E-12
VIM	1.01838079	0.651	0.39	1.64E-12
YBX1	0.53055372	0.949	0.854	2.33E-12
RPL6	0.40043821	1	0.988	2.78E-12
APOBEC3G	0.26512334	0.121	0.01	3.63E-12
PPIA	0.45755251	0.977	0.949	5.18E-12
RPL41	0.40876842	1	0.984	9.35E-12
EEF1A1	0.37607345	1	0.998	1.10E-11
IFNG	2.42783618	0.205	0.069	1.79E-11
CISH	0.27213271	0.191	0.039	2.07E-11
NCL	0.50475565	0.94	0.892	2.17E-11
IER3	0.82091044	0.642	0.371	2.71E-11

CYTOR	0.56174918	0.344	0.091	2.90E-11
TBX21	0.54564065	0.4	0.112	3.91E-11
RPS12	0.44211573	1	0.975	4.21E-11
MT.ND1	0.37084613	1	0.998	4.40E-11
RPS3A	0.42164553	1	0.979	4.80E-11
SLC7A5	0.56118287	0.647	0.442	6.48E-11
CCL3	1.08068476	0.065	0.009	7.27E-11
RPS27A	0.39466837	1	0.991	7.79E-11
RPS27	0.39432235	1	0.992	9.05E-11
APOBEC3C	0.31379398	0.121	0.011	9.52E-11
ATXN1	0.4125093	0.242	0.066	1.27E-10
RPL7	0.443437	0.981	0.917	1.37E-10
TRMT112	0.41456017	0.526	0.281	2.31E-10
RPLP1	0.43260384	0.958	0.905	2.99E-10
PRNP	0.56212877	0.665	0.458	4.05E-10
RPS3	0.45277348	0.926	0.819	4.68E-10
RPL37A	0.40404607	1	0.97	7.28E-10
RPS4X	0.48130723	0.995	0.92	7.48E-10
SAMSN1	0.56149574	0.544	0.313	9.96E-10
ZNF90	0.40733656	0.577	0.325	1.81E-09
NAMPT	0.77328115	0.735	0.52	2.38E-09
CFLAR	0.6137928	0.823	0.591	3.31E-09
ATP1B3	0.50893291	0.581	0.35	4.53E-09
NAP1L1	0.40336333	0.991	0.949	5.23E-09
KCNK5	0.16717097	0.116	0.015	7.36E-09
ENO1	0.45880546	0.935	0.848	7.50E-09
CYCS	0.4680815	0.847	0.77	8.93E-09
MT.ATP6	0.34177897	1	0.999	9.94E-09
STK24	0.31489917	0.247	0.083	1.53E-08
RPS21	0.41361322	0.977	0.896	1.54E-08
CD82	0.38683963	0.293	0.109	1.62E-08
GAPDH	0.46044322	0.553	0.308	1.75E-08
RPS28	0.43413093	0.916	0.806	2.05E-08
TNF	1.08581562	0.409	0.174	2.58E-08
RPS24	0.38580888	0.981	0.964	2.94E-08
PKM	0.48359244	0.907	0.826	6.83E-08
ZFP36L1	0.69014472	0.805	0.731	8.04E-08
MDFIC	0.53323077	0.6	0.355	1.13E-07
IRF2BP2	-0.5322223	0.349	0.582	1.13E-07
LAPTM4B	0.21451445	0.172	0.036	1.38E-07
LRIG1	0.22438831	0.158	0.034	1.54E-07
TMSB4X	-0.603295	0.856	0.93	1.68E-07
RPS20	0.35562436	0.995	0.98	2.05E-07
RGCC	0.60303062	0.609	0.425	2.28E-07
RPL5	0.36230651	0.991	0.968	2.57E-07

Table S2

SACS	0.48274329	0.577	0.309	3.88E-07
CDK6	0.59979788	0.563	0.337	4.65E-07
ARHGDIB	-0.4951369	0.172	0.411	5.20E-07
PARK7	0.37081218	0.507	0.29	5.92E-07
MTRNR2L8	0.33501939	0.521	0.365	6.40E-07
RPL34	0.3513544	1	0.986	1.34E-06
NBEAL1	0.40682923	0.656	0.526	1.44E-06
RPL11	0.37692217	0.995	0.946	1.54E-06
PPP1CC	0.37553109	0.447	0.283	2.82E-06
CD3D	0.41652727	0.581	0.408	2.97E-06
XBP1	0.43668003	0.623	0.342	3.12E-06
RPS8	0.38267574	1	0.968	3.53E-06
RPL31	0.35343918	1	0.977	3.99E-06
LRRFIP1	0.41110562	0.54	0.346	4.42E-06
TUBA1C	0.31104468	0.367	0.18	4.57E-06
TUBB	0.42508708	0.488	0.274	4.68E-06
CD3E	0.41796416	0.563	0.384	6.26E-06
NR4A1	-0.677923	0.414	0.555	7.74E-06
SEMA4D	0.27467509	0.247	0.099	8.25E-06
RPS13	0.30882593	1	0.988	8.69E-06
COPRS	0.19146662	0.116	0.028	9.78E-06
CD84	0.34669049	0.177	0.047	1.05E-05
RPL27	0.34874057	0.972	0.922	1.41E-05
RPL39	0.32680061	1	0.982	1.43E-05
CALR	0.43681218	0.53	0.351	1.49E-05
GEM	-0.740959	0.363	0.488	1.55E-05
F5	0.29543444	0.135	0.031	1.77E-05
CCT8	0.37178869	0.609	0.425	2.60E-05
EEF1B2	0.35303849	0.972	0.938	2.85E-05
RPS7	0.35166741	0.981	0.912	3.43E-05
TOMM7	0.39634813	0.753	0.579	3.97E-05
HNRNPAB	0.38621372	0.693	0.565	4.04E-05
SNRPF	0.31636246	0.447	0.247	4.69E-05
BTF3	0.34356033	0.926	0.761	4.99E-05
RPL21	0.30437834	0.995	0.991	5.40E-05
EXOC2	0.29481365	0.335	0.139	5.72E-05
BX890604.2	-0.3388955	0.051	0.254	6.02E-05
RPL23A	0.30918301	0.986	0.955	6.33E-05
CD200	0.89590263	0.6	0.261	6.67E-05
SLAMF1	0.55837826	0.488	0.204	6.88E-05
NME1	0.38015908	0.586	0.397	0.00010134
INSIG1	0.74065541	0.512	0.289	0.00010543
SGPP2	0.16349503	0.084	0.011	0.00011276
CRTAM	0.67438848	0.149	0.011	0.00011366
RPL38	0.34675142	0.977	0.896	0.00012027

Table S2

RBPJ	0.44136317	0.414	0.268	0.00012101
CHST11	0.18164862	0.144	0.03	0.00012232
RPL14	0.34543223	0.935	0.898	0.00012973
CCL3L1	0.42496415	0.051	0.003	0.0001332
HSP90B1	0.47328083	0.674	0.575	0.00015346
RPL32	0.31413776	1	0.98	0.00016342
SMCHD1	-0.4256057	0.27	0.459	0.00018858
ATM	-0.3608168	0.135	0.332	0.00028412
CERS2	0.26429921	0.279	0.124	0.0003234
RPL4	0.32460523	0.949	0.872	0.00036905
RPS15A	0.33150826	0.949	0.903	0.00039263
VMP1	0.4125161	0.553	0.346	0.00041234
SPRY1	0.61283754	0.349	0.085	0.0004614
RPLP2	0.34951443	0.888	0.8	0.00046634
SPCS2	0.28908392	0.349	0.219	0.0004733
GLUL	0.17866836	0.14	0.038	0.00048696
DNAJC3	0.28708093	0.209	0.104	0.00058476
PFKP	0.2506796	0.233	0.095	0.00060628
CLEC2B	-0.3616149	0.107	0.264	0.00070804
DDX21	0.32267282	0.986	0.947	0.00078135
TFRC	0.38707592	0.651	0.461	0.00080741
CREM	-0.4991119	0.633	0.726	0.0008145
PUS7	0.31829051	0.34	0.172	0.00086154
TAGAP	0.44694174	0.484	0.284	0.0008962
TOP1	0.36153672	0.651	0.514	0.00092775
MT.CO3	0.28093207	1	0.997	0.00107869
RPS11	0.34289459	0.809	0.701	0.0011362
MIR4435.2HG	0.21423761	0.13	0.023	0.00139046
ABLIM1	-0.3560797	0.13	0.304	0.00147939
MTRNR2L12	0.32687056	0.902	0.816	0.00170657
GBP5	0.71998895	0.66	0.385	0.00230477
ADAM19	0.19885388	0.13	0.032	0.00238631
RRP1B	0.24589454	0.307	0.145	0.00248421
NIPAL4	0.08830141	0.06	0.006	0.00256035
CD226	0.2197003	0.107	0.022	0.00258651
BTG2	-0.4211603	0.753	0.826	0.00267294
RPS5	0.36178066	0.907	0.808	0.00281864
NOP56	0.34591898	0.623	0.451	0.00283572
RPL19	0.31757026	0.884	0.787	0.00289839
SLC25A5	0.30735295	0.66	0.559	0.00295012
PPP1R18	0.3315262	0.423	0.267	0.00297613
AMIGO2	0.28320859	0.247	0.125	0.00325212
FRMD4B	0.1170881	0.065	0.006	0.00350441
CCT2	0.33742868	0.823	0.677	0.00511971
EGR3	-0.5618186	0.177	0.296	0.00547038

Table S2

RPL27A	0.38036642	0.902	0.79	0.00558135
RPL23	0.27876981	0.995	0.974	0.00562688
ZEB2	0.0968041	0.051	0.005	0.00633743
SEC61G	0.26927243	0.391	0.196	0.00639788
NCOA7	0.40717116	0.502	0.345	0.00681199
XCL2	0.33588999	0.033	0.002	0.00682642
HSPH1	0.35911584	0.628	0.464	0.00696678
TNFAIP8	0.4458074	0.833	0.579	0.00727415
NCS1	0.12585314	0.093	0.015	0.00736033
STAT4	0.28588064	0.26	0.124	0.00773559
GBP2	0.42106527	0.87	0.733	0.00897673
MSMO1	0.2848661	0.27	0.156	0.00919553
MALAT1	-0.3236432	0.981	0.988	0.00969074
CALM2	0.30691484	0.66	0.523	0.00986095
PDCD4	-0.3236357	0.195	0.366	0.01007615
NOLC1	0.33893961	0.833	0.744	0.01049243
STAMBPL1	0.12261457	0.07	0.01	0.0153637
SFXN1	0.50655846	0.558	0.314	0.01726372
CD44	0.33445664	0.981	0.936	0.01826508
SEMA7A	0.18211995	0.186	0.063	0.02059144
RPL9	0.24723599	0.995	0.989	0.0227567
HSPD1	0.36172782	0.809	0.618	0.02396607
RNF19A	0.39187594	0.409	0.231	0.02429099
CHSY1	0.38559816	0.595	0.415	0.02433046
PLEK	0.17436544	0.037	0.002	0.0244659
EIF4E	0.2592671	0.391	0.234	0.02582428
CSF2	0.15306881	0.042	0.003	0.02594697
GPR171	0.58065997	0.456	0.257	0.02677023
RPL36AL	0.27173131	0.595	0.439	0.02752894
WHAMM	-0.2695546	0.074	0.208	0.02777574
RPLP0	0.28823561	0.623	0.49	0.02924261
NHSL2	0.25687144	0.665	0.539	0.02982679
PSMB2	0.25151863	0.386	0.27	0.03019139
LTA	0.26824217	0.149	0.06	0.03104481
KPNB1	0.36181443	0.73	0.604	0.03168615
G3BP1	0.32423621	0.791	0.67	0.03188377
POLR1B	0.19941859	0.237	0.124	0.03243354
CFL1	0.31882112	0.842	0.74	0.03271519
SERBP1	0.29556895	0.795	0.728	0.03274823
EPOP	0.22397143	0.237	0.122	0.03279995
TPI1	0.27626154	0.316	0.197	0.03304489
RRAS2	0.16501501	0.126	0.052	0.03576515
SP140	0.17386401	0.144	0.044	0.03674321
PHLDA1	0.53364129	0.614	0.42	0.03688378
PSME3	0.2968398	0.642	0.476	0.03738256

Table S2

PAICS	0.30542563	0.684	0.53	0.03837203
SLC16A1	0.2742894	0.381	0.237	0.04091637
BCL2L1	0.17261846	0.158	0.057	0.04190908
PA2G4	0.31205763	0.712	0.565	0.04232952
GRPEL1	0.30972028	0.493	0.341	0.04300741
SLAMF7	0.19498773	0.06	0.008	0.04361495
RPL13A	0.27270066	0.986	0.952	0.04744998
VDR	0.06523402	0.051	0.005	0.04924696
**Unstimulated CD45RA$^-$ memory Tconv P1/HC**				
Gene	Average log_2(FC)	Patient % reads	Controls (n=2) % reads	Adjusted p-value
MT.ND3	0.79664877	0.996	0.995	1.68E-48
RPS26	1.00455673	0.731	0.281	3.42E-30
PABPC1	0.90110174	0.967	0.932	5.39E-26
TPT1	0.48490223	1	1	3.83E-18
MT.ND5	0.55081941	0.986	0.977	1.05E-16
MT.ND2	0.46906905	1	0.995	1.35E-14
MT.CYB	0.40593888	0.998	1	1.88E-10
HLA.C	0.5152003	0.934	0.76	1.88E-09
RPS16	0.46819457	0.938	0.842	2.58E-08
CD74 (HLA.DR) AbSeq	-1.2202097	0.971	0.982	9.84E-07
RPS3A	0.37172433	0.992	0.973	1.30E-06
MT.ATP6	0.3071909	0.998	0.995	8.07E-06
RPS12	0.35611542	0.99	0.968	1.35E-05
MT.ND1	0.30639035	0.998	0.991	2.81E-05
EEF1A1	0.33065971	0.998	0.995	0.00028042
RPL37	0.3345126	0.994	0.982	0.00054379
MT.CO2	0.26659362	0.992	0.995	0.00118392
MT.ND4	0.26190779	0.998	1	0.00260473
RPS27A	0.26619792	1	0.995	0.00316743
RPL10A	0.35026828	0.896	0.837	0.00599892
RPS29	0.24547446	1	0.995	0.00657313
S1PR1	0.36404771	0.6	0.489	0.00863636
RPS23	0.2919371	0.99	0.964	0.01526154
MT.CO1	0.23379154	1	1	0.01880477
MT.ND4L	0.30343803	0.952	0.937	0.04017553
**Stimulated CD45RA$^-$ memory Tconv P1/HC**				
Gene	Average log_2(FC)	Patient % reads	Controls (n=2) % reads	Adjusted p-value
RPS26	0.87629372	0.83	0.486	1.09E-27
MT.ND3	0.63313952	1	0.996	3.50E-26
HLA.C	0.70253517	0.935	0.78	1.98E-18
TUBB	0.63535096	0.514	0.255	6.29E-10
RPS16	0.46448734	0.971	0.934	2.83E-07

Table S2

TPT1	0.36089467	1	1	1.36E-06
NDFIP2	0.47334677	0.493	0.297	2.32E-05
MT.ND2	0.32308847	1	1	3.88E-05
IL32	0.53343542	0.446	0.239	4.05E-05
RPS27A	0.32283712	0.996	0.985	7.23E-05
STARD4	0.51389348	0.475	0.263	0.00012494
MT.CYB	0.31803214	1	0.996	0.00044778
HSPA5	0.64199378	0.837	0.73	0.00052562
TUBA1B	0.44560096	0.685	0.444	0.00098888
B2M	0.26924664	1	1	0.0025367
IL3	0.26863353	0.007	0.012	0.00390776
RPS12	0.29494959	1	1	0.01418338
PPIA	0.30882176	0.989	0.961	0.01846865
CYSLTR1	0.37344481	0.254	0.097	0.0230222
RPS11	0.34909443	0.826	0.722	0.02322863
RPS27	0.24988588	1	0.996	0.04939569

Table S3

Helios variant	p.Arg106Trp	p.Gly136_Ser191dup	p.Gly153Arg	p.Tyr200* (Daughter)	p.Tyr200* (Father)	p.Asn220Ser	p.Arg291*	p.Ile325Val	p.Val347Met	p.Tyr359Cys (Daughter)	p.Tyr359Cys (Mother)
Zygosity	Het.	Het.	Het.	Het.	Het.	Het.	Het.	Homo.	Het.	Het.	Het.
Consanguinity	—	—	—	ND	ND	ND	—	+	—	—	—
Functional impact	LOF*	DN	DN	LOF*	LOF*	LOF*	LOF*	LOF	None	None	None
Impact on protein expression	None	↓	None	Truncated	Truncated	None	Truncated	None	None	None	None
Developmental abnormalities	—	+	+	—	—	—	—	+	—	—	—
Clinical phenotype											
Infections	+	+	+	+	+	—	—	+	+	—	—
Mononucleosis	+ (EBV)	—	—	—	—	—	—	—	—	—	—
Recurrent (Sino)pulmonary/otitis media	+	+	+	+	+	—	—	+	+	—	—
Recurrent *C. albicans* (oral/vaginal)	—	—	—	+	+	—	—	—	—	—	—
Recurrent diarrhea	—	—	—	+	—	—	—	—	+	—	—
Recurrent stomatitis	—	—	—	—	—	—	—	—	+	—	—
Serositis									+	—	—
Lymphoma	—	—	—	—	+	—	—	—	—	—	—
(Focal segmental) glomerulonephritis	—	—	—	—	—	—	—	—	+	—	+
Immune phenotype											
CD3	↑	↓	↓	Normal	↓	Normal	Normal	↑	↓	ND	ND
CD4	↑	↑	↓	Normal	Normal	Normal	↓	↓	↓	↓	↓
Naive	Normal	↓	ND	Normal	↓	ND	Normal	Normal	↓	Normal	Normal
EM	Normal	Normal	ND	Normal	↑	ND	Normal	Normal	↑	↓	Normal
CM	Normal	↑	ND	↑	↑	ND	Normal	Normal	Normal	Normal	Normal
TEMRA	Normal	Normal	ND	Normal	Normal	ND	↓	Normal	Normal	Normal	Normal
Tregs	Normal	Normal	ND	↓	↓	ND	Normal	↓	↑	Normal	Normal
T_H1	ND	↑	ND	↑	↑	ND	ND	Normal	ND	ND	ND
T_H2	ND	↑	ND	ND	ND	ND	ND	Normal	ND	ND	ND
T_H9	ND	↑	ND	ND	ND	ND	ND	ND	ND	ND	ND
T_H17	ND	↑	ND	ND	ND	ND	ND	Normal	ND	ND	ND
cT_{FH}	ND	↑	ND	↓	↓	ND	ND	Normal	ND	ND	ND
T_H22	ND	Normal	ND	ND	ND	ND	ND	ND	ND	ND	ND
CD8	↑	↓	↓	↑	↑	↓	Normal	↑	Normal	↑	↑
Naive	Normal	↓	ND	↓	↓	Normal	Normal	↓	↓	Normal	Normal
EM	Normal	Normal	ND	Normal	Normal	Normal	Normal	↑	↑	Normal	Normal
CM	Normal	↑	ND	↑	Normal	Normal	Normal	Normal	Normal	Normal	Normal
TEMRA	Normal	Normal	ND	↑	↑	Normal	Normal	Normal	↓	↓	↓
$CD4^-CD8^-\gamma\delta^+$	ND	ND	ND	↓	↓	ND	ND	↑	ND	ND	ND
$CD4^-CD8^-\alpha\beta^+$	ND	ND	ND	↓	↓	ND	ND	↓	ND	ND	ND
MAIT	ND	ND	ND	↓	↓	ND	ND	↓	ND	ND	ND
NK	Normal	↑	ND	Normal	↑	↓	↓	↓	Normal	Normal	↓
CD19	↑	Normal	↓	Normal	Normal	Normal	Normal	↓	ND	ND	ND
Naive	Normal	Normal	ND	Normal	↑	Normal	Normal	↑	ND	ND	ND
Non-switched memory	↑	↑	ND	Normal	↓	↑	Normal	↓	ND	ND	ND
Switched memory	Normal	Normal	ND	↓	Normal	Normal	Normal	↓	ND	↓	Normal
Transitional	ND	↓	ND	↑	↑	ND	ND	↑	ND	ND	ND
Plasmablast	ND	Normal	ND	↓	↓	ND	ND	↓	ND	ND	ND
Autoantibodies	—	+	ND	+	+	—	+	—	—	+	+
Immunoglobulins											

Table S3

IgG	Normal	IVIG	↓	↓	Normal	ND	↑	↓	↓	ND	ND
IgA	↓	Normal	Normal	Normal	Normal	ND	ND	↓	↓	ND	ND
IgM	Normal	Normal	Normal	↓	Normal	ND	ND	↓	↓	ND	ND
IgE	ND	↑	ND	↑	Normal	ND	ND	Normal	ND	ND	ND
Vaccine responses	ND	Normal	↓	↓	Normal	ND	ND	↓	ND	ND	ND
Diptheria	ND	Normal	ND	↓	Normal	ND	ND	↓	ND	ND	ND
Tetanus	ND	Normal	↓	↓	Normal	ND	ND	↓	ND	ND	ND
S. pneumoniae	ND	ND	↓	↓	Normal	ND	ND	ND	ND	ND	ND
H. influenzae	ND	ND	↓	ND	Normal	ND	ND	ND	ND	ND	ND
Immune dysregulation/ autoimmunity	+	+	—	+	+	+	+	+	+	+	+
HLH	+	—	—	—	—	—	—	—	+	—	—
Still's disease/arthritis/joint pain	+	—	—	—	—	—	+	+	+	—	—
AIHA	—	+	—	—	—	+ (Evan's)	—	—	—	—	—
ITP	—	—	—	—	—	+ (Evan's)	+	—	—	+	—
Eczema	—	+	—	—	—	—	—	—	—	—	—
Lichen planus	—	—	—	—	+	—	—	—	—	—	—
Chronic lymphadenopathy	—	—	—	+	+	—	—	—	—	—	—
Vitiligo	—	—	—	+	—	—	—	—	—	—	—
Colitis	—	—	—	—	+	—	—	—	—	—	—
Hypothyroidism	—	—	—	+	+	—	—	+	—	—	—
SLE	—	—	—	—	—	—	+	—	—	+	+
Reference	Shahin	Lu	Mohajeri	Hetemaki	Hetemaki	Shahin	Shahin	Shahin	Shahin	Shahin	Shahin

Table S4

Phenotype	Frequency
Heterozygous *IKZF2* variant	10/13 (77%)
Recurrent sinopulmonary infections	7/13 (54%)
Immune dysregulation	10/13 (77%)
↓ % CD4	6/11 (55%)
Normal % CD4 subsets	6/9 (67%)
Normal % Tregs	5/9 (56%)
↑ % CD8	6/11 (55%)
↓ naïve, ↑ memory CD8	5/10 (50%)
↓ % MAIT	3/3 (100%)
Normal % CD19	5/8 (63%)
Autoantibodies	6/10 (60%)

Table S5

Target	Clone	Fluorophore	Catalogue #	Supplier
**Panel 1**				
**Target**	**Clone**	**Fluorophore**	**Catalogue #**	**Supplier**
CD3	UCHT1	BV510	300448	BioLegend
CD4	SK3	APC-R700	566909	BD Biosciences
CD8	RPA-T8	BV421	562428	BD Biosciences
CD25	2A3	BV786	741035	BD Biosciences
CD56	NCAM16.2	BUV661	750478	BD Biosciences
CD127	HIL-7R-M21	PE-CF594	562397	BD Biosciences
CD279 (PD-1)	EH12.1	PE-Cy7	561272	BD Biosciences
Helios	22F6	PE	137216	BioLegend
FOXP3	236A/E7	AF488	561181	BD Biosciences
IL-2	MQ1-17H12	BV650	564166	BD Biosciences
IL-10	JES3-19F1	BB700	566568	BD Biosciences
Fixable Viability	-	575V	565694	BD Biosciences
**Panel 2**				
**Target**	**Clone**	**Fluorophore**	**Catalogue #**	**Supplier**
CD3	UCHT1	BV510	300448	BioLegend
CD4	SK3	BUV563	612912	BD Biosciences
CD25	2A3	BUV805	742070	BD Biosciences
CD127	HIL-7R-M21	APC-R700	565185	BD Biosciences
FOXP3	236A/E7	AF488	561181	BD Biosciences
Helios	22F6	PE	137216	BioLegend
IL-4	MP4-25D2	PE-CF594	565161	BD Biosciences
IL-9	MH9A3	BV421	564254	BD Biosciences
IL-17A	N49-653	PerCP-Cy5.5	560799	BD Biosciences
IL-21	3A3-N2.1	AF647	560493	BD Biosciences
IL-22	2G12A41	PE-Cy7	366708	BioLegend
IFN-γ	4S.B3	BV711	564793	BD Biosciences
TNF-α	MAb11	BUV395	563996	BD Biosciences
Fixable Viability	-	575V	565694	BD Biosciences
**Panel 3**				
**Target**	**Clone**	**Fluorophore**	**Catalogue #**	**Supplier**
CD3	UCHT1	BV510	300448	BioLegend
CD4	SK3	APC-R700	566909	BD Biosciences
CD8	HIT8a	BV570	624298	BD Biosciences
CD19	HIB19	BUV737	741829	BD Biosciences
CD27	O323	BV605	751673	BD Biosciences
CD38	HIT2	APC	555462	BD Biosciences
CD45RA	HI100	BV421	562885	BD Biosciences
IgD	IA6-2	BUV615	613008	BD Biosciences
IgM	G20-127	BB515	564622	BD Biosciences
IL-2	MQ1-17H12	BV650	564166	BD Biosciences
IFN-γ	4S.B3	BV711	564793	BD Biosciences
TNF-α	MAb11	BUV395	563996	BD Biosciences
Helios	22F6	PE	137216	BioLegend

Table S5

Fixable Viability	-	780	565388	BD Biosciences
**Panel 4**				
**Target**	**Clone**	**Fluorophore**	**Catalogue #**	**Supplier**
CD3	SK7	APC-H7	560176	BD Biosciences
CD16	3G8	BV510	563830	BD Biosciences
CD27	O323	BV605	751673	BD Biosciences
CD56	NCAM16.2	BUV661	750478	BD Biosciences
CD57	NK-1	FITC	555619	BD Biosciences
CD94	HP-3D9	BUV805	748786	BD Biosciences
Helios	22F6	PE	137216	BioLegend
IFN-γ	4S.B3	BV711	564793	BD Biosciences
TNF-α	MAb11	BUV395	563996	BD Biosciences
Perforin	δG9	PE-CF594	563763	BD Biosciences
Granzyme B	GB11	AF647	561999	BD Biosciences
Fixable Viability	-	575V	565694	BD Biosciences
**Panel 5**				
**Target**	**Clone**	**Fluorophore**	**Catalogue #**	**Supplier**
CD4	RPA-T4	FITC	555346	BD Biosciences
CD25	BC96	APC	567316	BD Biosciences
CD127	HIL-7R-M21	BV421	562436	BD Biosciences
Fixable Viability	-	780	565388	BD Biosciences
**Panel 6**				
**Target**	**Clone**	**Fluorophore**	**Catalogue #**	**Supplier**
CD3	UCHT1	APC	300412	BioLegend
CD4	OKT4	PE	317409	BioLegend
CD8	HIT8a	FITC	300906	BioLegend
CPD	-	eF450	65-0842-85	ThermoFisher
Fixable Viability	-	eF780	65-0865-14	ThermoFisher
**Panel 7**				
**Target**	**Clone**	**Fluorophore**	**Catalogue #**	**Supplier**
CD4	OKT4	BV421	317434	BioLegend
CD14	61D3	APC-eF780	47-0149-42	ThermoFisher
CD25	4E3	PE	130-113-282	Miltenyi Biotec
CD127	HIL-7R-M21	R718	566967	BD Biosciences
Fixable Viability	-	eF780	65-0865-14	ThermoFisher
